# Geochemical Characteristics and Formation Environment of Mudstones in the Xueyuanhe Formation in the Raggyorcaka Area, Qiangtang Basin

**DOI:** 10.1021/acsomega.6c02947

**Published:** 2026-06-15

**Authors:** Jie Peng, Yuan Xie, Mei Yue, Qinghua Peng, Zhongwei Wang, Rui Wang, Zhonglin Wang, Yonghong Hu

**Affiliations:** † Civil-Military Integration Center of China Geological Survey, Chengdu, Sichuan 610036, China; ‡ College of Energy, 47908 Chengdu University of Technology, Chengdu, Sichuan 610059, China; § School of Geoscience and Technology, Southwest Petroleum University, Chengdu, Sichuan 610500, China; ∥ Oil & Gas Survey Center of China Geological Survey, Beijing 100083, China

## Abstract

The Permian source rocks in the Qiangtang Basin exhibit good potential for oil and gas exploration. Elucidating their organic matter enrichment mechanism and sedimentary paleoenvironmental characteristics is of great significance for evaluating the basin’s Paleozoic oil and gas resources. This paper presents a systematic study of the Middle Permian mudstones from the Xueyuanhe Formation in the Raggyorcaka area of the North Qiangtang Depression (NQD), integrating petrological, organic geochemical, and elemental geochemical analyses. The key objectives are to characterize the hydrocarbon source rock potential, paleoenvironment, provenance, and tectonic setting of these mudstones, and thereby reconstruct their organic matter enrichment mechanism. The total organic carbon and chloroform bitumen “A” contents of these mudstones are in the range of 0.42% to 0.60% (avg. of 0.52%) and 0.006–0.014% (avg. of 0.009%), respectively. They are interpreted as poor source rocks. Kerogen index (TI), kerogen H/C and O/C atomic ratios indicate that the organic matter type is predominantly type II_2_ kerogen, with minor type III kerogen. Vitrinite reflectance (Ro) and pyrolysis peak temperature (*T*_max_) of samples exhibit a high degree of thermal evolution. Medium chemical index of alteration (CIA) values of samples indicates a warm and humid climate. Redox indexes (V/Cr, Ni/Co, U/Th, Mo_EF_–U_EF_) reveal that these mudstones were deposited in oxic conditions. Low Sr/Ba and Ca/(Ca+Fe) ratios indicate a low-salinity water column during the Xueyuanhe Formation mudstones deposition. Fe/Ti–Al/(Al + Fe + Mn) diagram and Al–Fe–Mn ternary diagram indicate that these mudstones were not affected by hydrothermal activity. Terrigenous flux proxies (Al, Zr, Ti_EF_, and Zr_EF_) indicate that there was less input of terrigenous detritus during sedimentation period. Relatively low Si_bio_, Ba_bio_, and P/Ti values reveal that the level of primary productivity in the surficial water column was relatively low. Analysis of sediment provenance and tectonic setting reveals that the Xueyuanhe Formation mudstones are derived mainly from felsic volcanic rocks, with possible minor inputs from basaltic and sedimentary sources, the source area corresponding to a continental island arc environment. The warm and humid paleoclimate during deposition of the Xueyuanhe Formation favored organic matter preservation. However, the combination of low primary productivity, oxic water conditions, slow sedimentation rates, and continuous input of felsic terrigenous detritus inhibited the enrichment of organic matter, ultimately resulting in the poor hydrocarbon generation potential of these mudstones.

## Graphical abstract

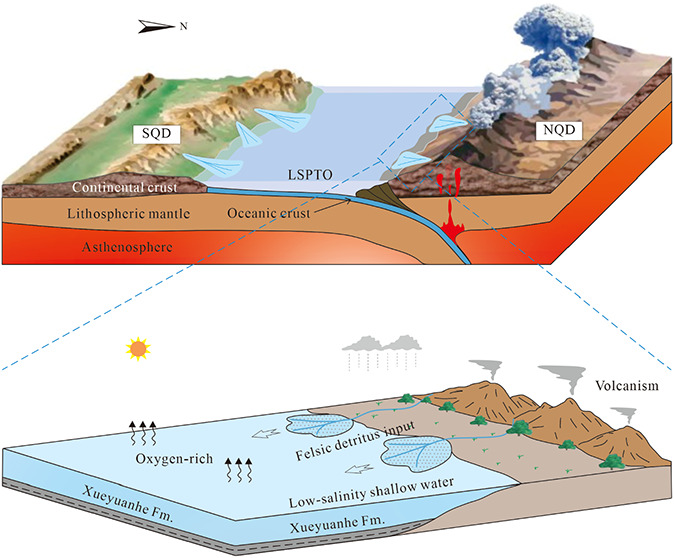

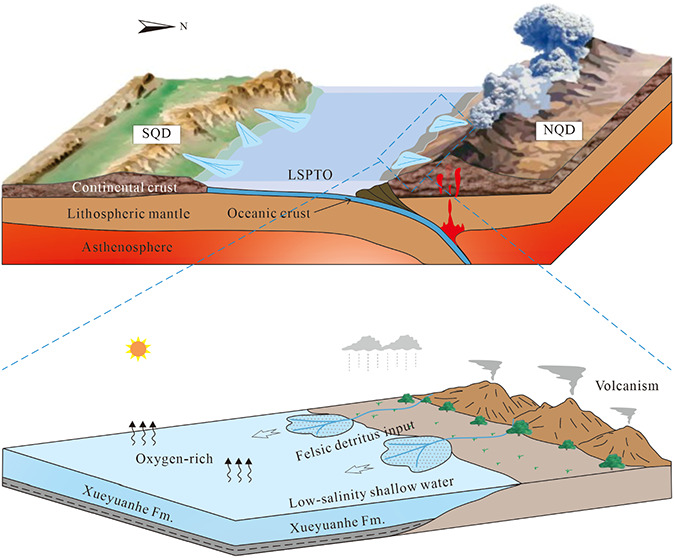

## 1 Introduction

The Tethys tectonic domain is of paramount importance as a global oil and gas concentration area, and its hydrocarbon occurrence conditions and exploration prospects have long attracted worldwide attention.^1,2^ The Qiangtang Basin, located in the eastern segment of the Tethys tectonic domain, is a Mesozoic marine sedimentary basin. It represents the largest new frontier for onshore oil and gas exploration in China and is characterized by the most complete stratigraphic succession and a relatively low exploration maturity.^3–5^ As the Paleo-Tethys Ocean continued its northward subduction at the end of the Paleozoic, coupled with a global sea-level drop, the Qiangtang Basin experienced regional uplift and a subsequent large-scale marine regression.^6,7^ During the Permian, a thick succession of shallow marine carbonate rocks (platform facies) and marine-continental transitional coal-bearing formations formed in the North Qiangtang Depression (NQD).^8^ Simultaneously, multiple intervals of organic-rich source rocks were developed within this sedimentary succession. These coeval successions are mainly represented by the Zhanjin Formation in the Lower Permian, the Xueyuanhe Formation in the middle Permian, the Nayixiong Formation in the Upper Permian, and the Raggyorcaka Formation in the Upper Permian. The Zhanjin Formation comprises mainly slope facies and occur in the southwest of the NQD.^5,9,10^ The Nayixiong Formation was deposited in deltaic-swamp environments and occurred in the east of the NQD.^7,11,12^ The Xueyuanhe Formation and Raggyorcaka Formation were deposited in marine-continental transitional facies and occur in the southwest of the NQD.^13,14^ Through geochemical, sedimentological, and biostratigraphic analyses, previous studies have conducted preliminary investigations on these source rocks and have demonstrated that the Permian source rocks in the Qiangtang Basin possess significant hydrocarbon generation potential, making them an important exploration target for the next phase of oil and gas exploration.^12,15^

A succession of dark gray to gray-black mudstones, tens of meters in thickness, has recently been identified in the Middle Permian Xueyuanhe Formation along the southwestern margin of the NQD. The hydrocarbon generation potential and depositional paleoenvironment of these mudstones remain unreported in the literature. Therefore, this paper takes these mudstones of the Xueyuanhe Formation in the Raggyorcaka area of the NQD as the research object. Based on organic geochemical data, the organic matter abundance, type and maturity of the Xueyuanhe Formation mudstones are evaluated, and their hydrocarbon generation potential is identified. Building on these organic geochemical results, this study integrated petrological and elemental geochemical data to reconstruct the paleoenvironmental conditions during deposition, including climate, salinity, redox conditions, and primary productivity. In addition, the sediment provenance and tectonic setting of the Xueyuanhe Formation mudstones were also constrained. Based on these findings, the formation mechanism of these mudstones was elucidated, and a corresponding sedimentary model was established, providing scientific support for the evaluation of the Paleozoic oil and gas resources in the Qiangtang Basin.

## 2 Geological Background

The Qiangtang Basin is in the central part of the Tibetan Plateau, with an area of approximately 18.5 × 10^4^ km^2^. It is bordered to the north by the Jinshajiang Suture Zone (JSSZ) and to the south by the Bangong-Nujiang Suture Zone (BNSZ). Based on geophysical characteristics and regional geological survey data, the Qiangtang Basin can be divided into three secondary tectonic units from north to south: the North Qiangtang Depression (NQD), the Central Uplift Belt (CUB), and the South Qiangtang Depression (SQD) (Figure 1a).^16–18^

**Figure 1:**
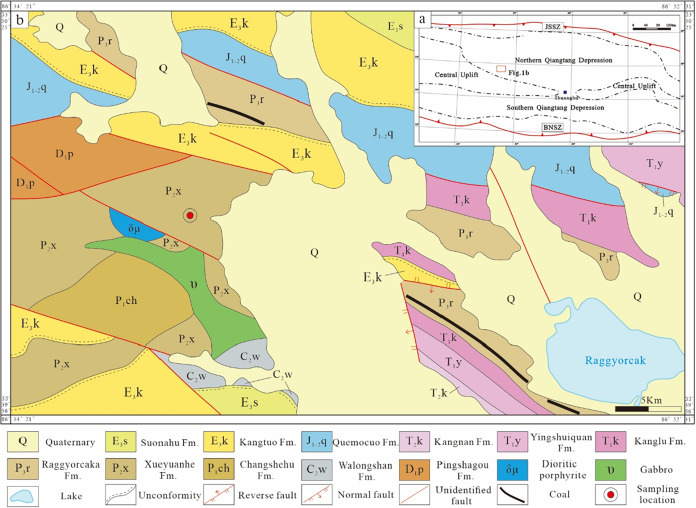
Location and geological context of the Xueyuanhe Formation in the Qiangtang Basin. (a) Regional location; (b) Geologic map of the Raggyorcaka area.

The Paleozoic was a crucial period in the evolution of the Qiangtang Basin. From the Devonian to the end of the Permian, the basin recorded a transition from prerift, through early rifting and main rifting, to compressional filling stages. Correspondingly, the stratigraphic succession comprises carbonate formations (D), continental clastic formations (C–P_1_), continental volcanic formations (C), marine volcanic-sedimentary formations (P_1_), and marine-continental transitional coal-bearing clastic formations (P_2_).^19^

The study area is located at the southwestern margin of the NQD, adjacent to the CUB. The exposed stratigraphic succession in this area comprises, from bottom to top: the Devonian Strata (Pingshagou Formation), the Carboniferous Strata (Walongshan Formation), the Permian Strata (Changshehu Formation, Xueyuanhe Formation, Raggyorcaka Formation), the Triassic Strata (Kanglu Formation, Yingshuiquan Formation, Kangnan Formation), the Jurassic Strata (Quemocuo Formation), and the Paleogene Strata (Kangtuo Formation, Suonahu Formation) (Figure 1b). Regional fault structures are well-developed in the area, predominantly trending NW. Fold structures are observed on the western shore of Raggyorcaka Lake, involving Permian and Triassic strata with NW-trending axes and continuous deformation. Both ends of these folds are covered by Quaternary strata, and the exposed axial length is approximately 13 km. The Xueyuanhe Formation was first established in the Regional Geological Survey Report of the People’s Republic of China (Mayigangri Map, I45C003002).^20^ It is mainly exposed approximately 15 km west of Raggyorcaka, consisting of medium- to thin-bedded micrite intercalated with calcarenite, bioclastic limestone and fine-grained detrital rock. Fossilized fusulinids and foraminifera are commonly present in the limestone. The base of Xueyuanhe Formation is conformable contact with the Lower Permian Changshehu Formation, while the upper part is conformable contact with the Raggyorcaka Formation.^21^

## 3 Samples and Analytical Methods

This study conducted detailed stratigraphic measurements and continuous sampling on the mudstone outcrops of the Xueyuanhe Formation. A total of 30 samples were collected from bottom to top at intervals of less than 1 m, with samples excavated more than 25 cm below the weathered surface. The sampling site is located at 86°39′26.11″E, 33°44′55.77″N, at an elevation of 4887 m. All analyses, including total organic carbon (TOC), chloroform bitumen “A”, Rock-Eval pyrolysis, kerogen elemental composition, maceral composition, vitrinite reflectance (Ro), major and trace elements, rare earth elements, and petrographic thin sections, were performed at the Sichuan Keyuan Testing Center of Engineering Technology. Petrographic thin sections were prepared using a JKPG-250 apparatus and in accordance with the standard DZ/T 0275.2–2015.

Samples for TOC were first treated with phosphoric acid to remove inorganic carbon before TOC values were measured using a carbon–sulfur analyzer (CS230) with a lower detection limit of 100 μg/g (0.01%) in the low-carbon mode. The detection range in low-carbon mode was 10 ppm, with an absolute analysis error of 20 ppm. The measurement process complies with Chinese national standards GB/T 19145–2022. Bitumen from 20 selected bulk powdered samples (120 mesh) were extracted by using a solvent mixture of dichloromethane and methanol in Soxhlet apparatus for 72 h and then dried and weighed to obtain asphalt contents representing soluble bitumen in mudstones. For Rock-Eval analysis, 20 bulk powdered samples (120 mesh) were placed in helium gas and heated for 3 min at 300 °C to measure *S*_1_ values (free hydrocarbon abundance; mg HC/g rock) and linearly heated to 600 °C with a constant rate (25 °C/min) to obtain *S*_2_ (hydrocarbon abundance generated from organic matter pyrolysis; mg HC/g rock), *S*_3_ (the volume of CO_2_ generated after heating to 390 °C; mg CO_2_/g rock), and *T*_max_ (the temperature when *S*_2_ values reach its maximum; °C) values. Rock-Eval pyrolysis was conducted using a Rock-Eval YQ-VIIA instrument, following the Chinese industry standard GB/T 18602–2012.

The extracted kerogen must undergo cleaning, freezing, and freeze-drying processes before it can be subjected to elemental analysis. Kerogen was isolated from 10 samples for maceral analysis under a Leica DM4500P polarizing microscope and for stable carbon isotope measurement using a DELTA V isotope ratio mass spectrometer, adhering to Chinese industry standards GB/T 19144–2010 and SY/T 6414–2014. Vitrinite reflectance (Ro) was measured on 10 kerogen samples using a Zeiss Axio Scope A1/J&M MSP 200 microspectrophotometer with a 50 × 85 oil immersion objective at a wavelength of 546 nm, more than 50 points were measured on each sample, following the Chinese industry standard GB/T14506.30–2010.

For major element analysis, 0.6 g of each bulk powder sample (<200 mesh) and 6 g lithium metaborate flux were mixed in a Pt–Au crucible, and then fused in a high-frequency melting furnace at 1050 °C for 11 min. The completely melted sample was poured into a mold to form a thin flat surfaced glass disk and then determined by AxiosMAX X fluorescence spectrometer with a relative analysis error of ±5%. For trace and rare earth elemental analysis, 50 mg powder sample (<200 mesh) was dissolved in a Teflon bomb using 0.5 mL HNO_3_ and 1 mL HF at a constant temperature (185 °C) for 24 h to dry. The dried powder was then treated with 0.5 mL HNO_3_. The powder sample was acidified twice and then treated using 5 mL HNO_3_ at a constant temperature (130 °C) for 3 h. Dissolved sample in PET bottle was diluted to 50 mL for further analyzed by ICAP RQ inductively coupled plasma mass spectrometer. The analytical precision of the major and trace elements was all better than 5%, following the Chinese industry standard GB/T 14506.30–2010.

## 4 Results and Analysis

### 4.1 Petrologic Characteristics

Field geological route investigation reveals that the Xueyuanhe Formation is characterized by dark gray to black thin-bedded mudstones, interbedded with minor sandy mudstones. The bottom of the mudstone interval is covered, and its exposed thickness exceeds 20 m (Figure 2a,b). Microscopic observation shows that mudstone consists predominantly of microcrystalline clay mineral aggregates, with a minor amount of terrigenous detritus. The terrigenous detritus is mainly composed of fine sand and silt, with particle sizes ranging from 0.03 mm to 0.15 mm, scattered throughout the clay minerals. In addition, a small amount of carbonate minerals (calcite and dolomite) are present as fine crystals mixed within the clay minerals, with the crystal grains generally less than 0.02 mm (Figure 2c,d). Sandy mudstones are mainly composed of microcrystalline to cryptocrystalline clay mineral aggregates, with minor terrigenous detritus. The detrital component is predominantly quartz, accompanied by subordinate feldspar. Most grains are angular in shape, with particle sizes ranging from 0.03 mm to 0.06 mm and a maximum of 0.2 mm. They are predominantly distributed in band-like arrangements within the rock. In both mudstones and sandy mudstones, minor amounts of fine-grained pyrite and organic detritus are present, disseminated within the clay minerals.

**Figure 2:**
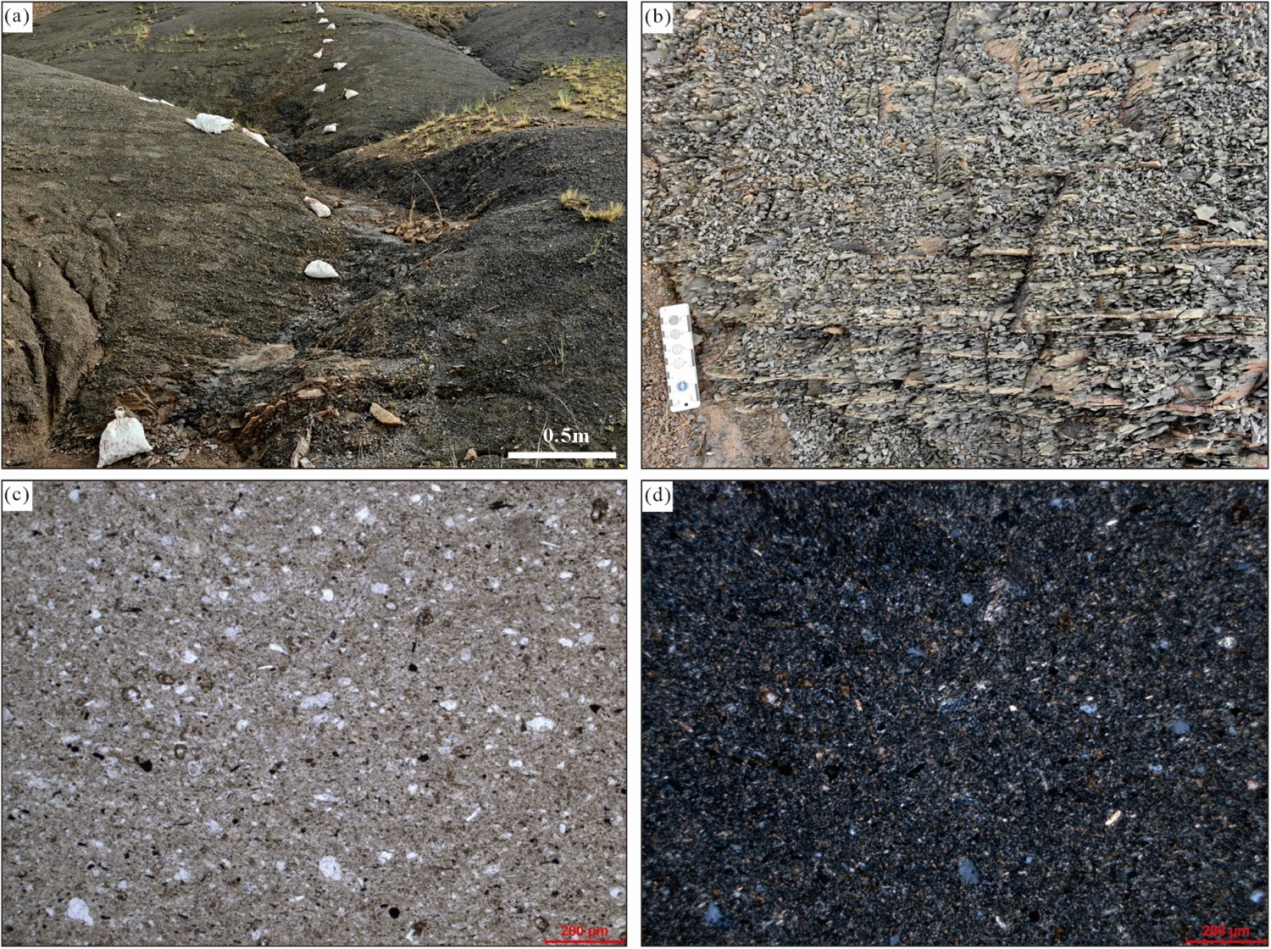
Outcrop photographs and microphotographs of mudstones from the Xueyuanhe Formation in Raggyorcaka area. (a) Macroscopic outcrop; (b) microscopic outcrop; (c) single polarized light photos; (d) orthogonal polarized light photos.

### 4.2 Organic Geochemical Characteristics

The sampling layers and analytical results, including TOC, Rock-Eval pyrolysis, chloroform bitumen “A”, vitrinite reflectance (Ro), for the Middle Permian Xueyuanhe Formation mudstones from the Raggyorcaka area are summarized in Table 1. The TOC content of the 30 samples ranges from 0.42% to 0.60% (avg. of 0.52%), and the TOC content is generally low and shows no significant variation along the vertical direction. Rock-Eval pyrolysis analyses of 30 mudstones yielded *S*_1_, *S*_2_, and *S*_3_ abundances of 0.01 mg/g–0.02 mg/g (avg. of 0.02 mg/g), 0.08 mg/g–0.12 mg/g (avg. of 0.1 mg/g), and 0 mg/g, respectively. The Rock-Eval pyrolysis peak temperature (*T*_max_) values of these samples are 524 °C–557 °C (avg. of 554 °C). The hydrogen index (HI) and oxygen index (OI) values of the analyzed samples are 14.39–23.44 mg HC/g TOC (avg. of 18.64 mg/g TOC) and 0 mg CO_2_/g TOC, respectively. The content of chloroform bitumen “A” in the sample is extremely low, ranging from 0.006% to 0.014% (avg. of 0.009%). The vitrinite reflectance (Ro) values of the samples ranging from 0.85% to 1.94% (avg. of 1.72%).

**Table 1:** Basic Organic Geochemical Parameters of Mudstones from the Xueyuanhe Formation in Raggyorcaka Area

				Rock-Eval pyrolysis					
Sample	Lithology	Depth (m)	TOC (%)	*S*_0_ (mg/g)	*S*_1_ (mg/g)	*S*_2_ (mg/g)	*T*_max_ (°C)	Chloroform bitumen “A”	Ro (%)
P_2_x-1	mudstone	0.8	0.55	0.01	0.02	0.11	557	0.011	1.70
P_2_x-2	mudstone	1.6	0.52	/	/	/	/	0.009	/
P_2_x-3	mudstone	2.5	0.52	0.01	0.02	0.12	524	0.007	/
P_2_x-4	mudstone	3.2	0.53	/	/	/	/	0.009	1.60
P_2_x-5	mudstone	4.1	0.53	0.01	0.02	0.08	557	0.008	/
P_2_x-6	mudstone	4.8	0.55	/	/	/	/	0.007	/
P_2_x-7	mudstone	5.7	0.52	0.01	0.01	0.08	556	0.008	/
P_2_x-8	mudstone	6.4	0.55	/	/	/	/	0.006	1.89
P_2_x-9	mudstone	7.3	0.60	0.01	0.01	0.09	557	0.010	/
P_2_x-10	mudstone	8	0.49	/	/	/	/	0.008	/
P_2_x-11	mudstone	8.9	0.56	0.01	0.01	0.08	557	0.010	/
P_2_x-12	mudstone	9.6	0.53	0.01	0.01	0.08	557	0.009	1.80
P_2_x-13	mudstone	10.5	0.42	0.01	0.02	0.08	556	0.011	/
P_2_x-14	mudstone	11.2	0.49	0.01	0.02	0.10	557	0.009	1.92
P_2_x-15	mudstone	12.1	0.50	0.01	0.02	0.09	557	0.008	/
P_2_x-16	mudstone	12.8	0.54	0.01	0.02	0.10	557	0.010	1.82
P_2_x-17	mudstone	13.7	0.51	0.01	0.02	0.12	554	0.010	/
P_2_x-18	mudstone	14.8	0.50	0.01	0.02	0.11	554	0.011	1.82
P_2_x-19	mudstone	15.7	0.54	0.01	0.02	0.11	555	0.013	/
P_2_x-20	mudstone	16.5	0.50	0.01	0.02	0.09	554	0.014	/
P_2_x-21	mudstone	17.3	0.47	/	/	/	/	0.011	1.94
P_2_x-22	mudstone	18.1	0.51	0.01	0.02	0.09	554	0.009	/
P_2_x-23	mudstone	18.9	0.47	/	/	/	/	0.007	/
P_2_x-24	mudstone	19.7	0.54	0.01	0.02	0.10	555	0.009	/
P_2_x-25	mudstone	20.5	0.53	/	/	/	/	0.008	1.84
P_2_x-26	mudstone	21.3	0.60	0.01	0.02	0.12	554	0.007	/
P_2_x-27	mudstone	22.1	0.53	/	/	/	/	0.008	/
P_2_x-28	mudstone	22.9	0.51	0.01	0.02	0.10	554	0.006	/
P_2_x-29	mudstone	23.8	0.53	/	/	/	/	0.010	/
P_2_x-30	mudstone	24.6	0.53	0.01	0.02	0.10	555	0.008	0.85

The mudstone samples in the study area have similar maceral composition and elemental composition of kerogen (Table 2). These include exinite (53%–73%, avg. of 65%), vitrinite (24%–45%, avg. of 32%), and inertinite (2%–4%, avg. of 3%). The analysis results of the organic element composition show that the C atom content of the samples ranges from 29.44%–78.23% (avg. of 52.31%), the H atom content ranges from 1.74%–3.82% (avg. of 2.71%), the O atom content ranges from 2.65%–6.38% (avg. of 4.19%), the H/C atom ratio ranges from 0.05–0.06 (avg. of 0.05), and the O/C atom ratio ranges from 0.07–0.09 (avg. of 0.08). The H/C and O/C atom ratios of all samples are extremely low and relatively close.

**Table 2:** Microscopic Components of Kerogen in Mudstones from the Xueyuanhe Formation in Raggyorcaka Area

				Organic element (%)			Microscopic components (%)				
Sample	Lithology	Depth (m)	TOC (%)	C	H	O	Exinite	Vitrinite	Inertinite	Type index	Type
P_2_x-1	mudstone	0.8	0.55	45.59	2.42	3.18	66	30	4	6.5	II_2_
P_2_x-4	mudstone	3.2	0.53	32.21	1.87	2.97	63	34	3	3	II_2_
P_2_x-8	mudstone	6.4	0.55	37.63	1.97	2.88	61	36	3	0.5	II_2_
P_2_x-12	mudstone	9.6	0.53	53.67	2.68	3.98	63	34	3	3	II_2_
P_2_x-14	mudstone	11.2	0.49	53.82	2.77	4.01	64	32	4	4	II_2_
P_2_x-16	mudstone	12.8	0.54	78.23	3.68	6.38	67	30	3	8	II_2_
P_2_x-18	mudstone	14.8	0.50	42.10	2.41	3.46	71	26	3	13	II_2_
P_2_x-21	mudstone	17.3	0.47	29.44	1.74	2.65	73	24	3	15.5	II_2_
P_2_x-25	mudstone	20.5	0.53	76.76	3.71	6.05	53	45	2	–9.25	III
P_2_x-30	mudstone	24.6	0.53	73.65	3.82	6.33	68	30	2	9.5	II_2_

### 4.3 Element Geochemical Characteristics

Major and trace element abundances of the Xueyuanhe Formation mudstone samples are listed in Tables 3 and 4. Major element analysis shows that SiO_2_, Al_2_O_3_, and Fe_2_O_3_ are the main oxides, with contents ranging from 58.82% to 61.30%, 16.14% to 17.12%, and 5.94% to 6.71%, respectively; the contents of K_2_O, MgO, and CaO are intermediate, ranging from 2.85% to 3.32%, 2.10% to 2.53%, and 1.50% to 2.98%, respectively; the contents of Na_2_O, P_2_O_5_, TiO_2_, and MnO have the lowest contents, ranging from 0.58% to 0.76%, 0.14% to 0.18%, 0.63% to 0.70%, and 0.06% to 0.14%, respectively (Table 3). Among the trace elements, Ti, Mn, and Ba exhibit the highest concentrations, with average values of 3699.26 μg/g, 726.09 μg/g, and 368.74 μg/g, respectively. The concentrations of V, Rb, Zr, Zn, Cr, and Sr are relatively lower, with average values of 150.04 μg/g, 142.56 μg/g, 137.14 μg/g, 116.40 μg/g, 115.78 μg/g and 103.21 μg/g, respectively. The concentrations of the remaining trace elements are all lower than 50 μg/g (Table 4).

**Table 3:** Major Elements Geochemical Parameters of Mudstones from the Xueyuanhe Formation in Raggyorcaka Area

			Major element (%)															
Sample	Depth (m)	TOC (%)	Al_2_O_3_	CaO	Fe_2_O_3_	MgO	MnO	P_2_O_5_	K_2_O	SiO_2_	Na_2_O	TiO_2_	CIA	Si_bio_	Ba_bio_	Zr/Al	Ti/Al	Fe/Ti
P_2_x-1	0.8	0.55	16.45	2.69	6.20	2.29	0.14	0.17	3.04	60.21	0.75	0.69	78.38	1.00	364.38	16.93	0.05	9.49
P_2_x-2	1.6	0.52	16.41	2.80	6.31	2.24	0.10	0.18	3.03	59.86	0.75	0.68	78.39	0.91	337.93	17.10	0.05	9.73
P_2_x-3	2.5	0.52	16.73	2.22	6.11	2.24	0.09	0.17	3.10	60.42	0.75	0.69	78.41	0.64	347.76	15.21	0.05	9.23
P_2_x-4	3.2	0.53	16.66	2.68	6.10	2.21	0.13	0.17	3.07	59.95	0.76	0.68	78.42	0.54	409.71	16.85	0.05	9.37
P_2_x-5	4.1	0.53	16.92	2.02	5.94	2.15	0.07	0.18	3.14	60.57	0.74	0.68	78.57	0.41	343.05	15.92	0.05	9.11
P_2_x-6	4.8	0.55	16.71	2.31	6.31	2.26	0.08	0.17	3.04	60.13	0.73	0.70	78.80	0.54	338.44	14.50	0.05	9.54
P_2_x-7	5.7	0.52	16.39	2.54	6.29	2.25	0.12	0.18	3.03	60.07	0.71	0.67	78.64	1.04	350.66	15.71	0.05	9.86
P_2_x-8	6.4	0.55	16.81	2.49	6.39	2.34	0.11	0.16	3.14	59.95	0.73	0.69	78.54	0.29	369.90	15.04	0.05	9.73
P_2_x-9	7.3	0.60	16.49	2.71	6.36	2.25	0.10	0.17	3.06	59.98	0.72	0.69	78.59	0.83	355.37	14.48	0.05	9.66
P_2_x-10	8	0.49	16.59	2.36	6.33	2.27	0.11	0.17	3.04	60.43	0.73	0.69	78.66	0.89	350.30	15.77	0.05	9.59
P_2_x-11	8.9	0.56	16.48	2.62	6.35	2.27	0.08	0.17	3.02	59.99	0.72	0.69	78.71	0.85	331.41	15.77	0.05	9.70
P_2_x-12	9.6	0.53	16.55	2.13	6.36	2.29	0.09	0.17	3.05	60.88	0.71	0.68	78.77	1.15	353.21	15.76	0.05	9.82
P_2_x-13	10.5	0.42	17.05	2.19	6.68	2.53	0.08	0.16	3.19	59.99	0.72	0.70	78.65	–0.08	370.40	14.90	0.05	10.02
P_2_x-14	11.2	0.49	16.94	2.71	6.04	2.30	0.09	0.18	3.15	60.59	0.75	0.69	78.46	0.38	343.75	14.98	0.05	9.19
P_2_x-15	12.1	0.50	16.47	2.78	6.49	2.35	0.11	0.18	3.01	60.11	0.72	0.68	78.76	0.94	393.12	15.62	0.05	9.95
P_2_x-16	12.8	0.54	16.76	2.41	6.46	2.26	0.08	0.18	3.09	60.75	0.75	0.69	78.52	0.75	371.82	15.17	0.05	9.79
P_2_x-17	13.7	0.51	17.12	1.50	6.71	2.34	0.06	0.18	3.19	61.30	0.71	0.70	78.78	0.41	357.76	16.34	0.05	10.13
P_2_x-18	14.8	0.50	16.36	1.96	6.42	2.26	0.09	0.18	3.00	60.91	0.69	0.68	78.89	1.48	406.23	16.36	0.05	9.91
P_2_x-19	15.7	0.54	17.01	2.25	6.25	2.18	0.07	0.15	3.29	59.49	0.63	0.66	78.90	–0.25	366.50	14.43	0.05	9.93
P_2_x-20	16.5	0.50	16.73	2.09	6.29	2.19	0.08	0.18	3.15	59.89	0.64	0.65	79.06	0.39	349.85	13.87	0.05	10.21
P_2_x-21	17.3	0.47	16.94	2.50	6.15	2.10	0.11	0.14	3.32	58.82	0.59	0.63	79.04	–0.44	428.57	16.13	0.05	10.20
P_2_x-22	18.1	0.51	16.30	2.30	6.37	2.16	0.09	0.14	3.09	59.62	0.58	0.63	79.29	0.98	390.54	15.66	0.05	10.61
P_2_x-23	18.9	0.47	16.60	1.55	6.36	2.22	0.06	0.15	3.17	60.18	0.59	0.63	79.26	0.75	385.63	15.02	0.05	10.52
P_2_x-24	19.7	0.54	16.83	1.82	6.45	2.29	0.07	0.15	3.16	59.93	0.63	0.67	79.24	0.26	390.80	14.59	0.05	10.16
P_2_x-25	20.5	0.53	16.61	2.57	6.33	2.23	0.10	0.16	3.09	59.32	0.64	0.66	79.16	0.34	404.99	15.49	0.05	10.02
P_2_x-26	21.3	0.60	16.58	2.56	6.47	2.28	0.06	0.16	3.05	59.08	0.65	0.68	79.22	0.28	364.11	15.49	0.05	10.04
P_2_x-27	22.1	0.53	16.69	2.28	6.32	2.23	0.09	0.16	3.06	59.36	0.65	0.68	79.27	0.22	351.50	15.90	0.05	9.78
P_2_x-28	22.9	0.51	16.14	2.98	6.11	2.24	0.10	0.16	2.85	59.40	0.70	0.68	79.13	1.15	409.06	16.72	0.05	9.41
P_2_x-29	23.8	0.53	16.36	2.59	6.07	2.23	0.08	0.16	2.97	60.02	0.69	0.68	79.04	1.06	331.34	15.60	0.05	9.31
P_2_x-30	24.6	0.53	16.29	2.82	6.03	2.15	0.10	0.17	2.94	60.01	0.68	0.67	79.08	1.17	393.14	16.03	0.05	9.45

**Table 4:** Trace Elements Geochemical Parameters of Mudstones from the Xueyuanhe Formation in Raggyorcaka Area

	Trace element (μg/g)																				
Sample	Sr	Cu	Ba	V	Cr	Zr	U	Mo	Ti	Ni	Co	C	U_EF_	Mo_EF_	Ti_EF_	Zr_EF_	Sr/Cu	Sr/Ba	V/Cr	Ni/Co	U/Th
P_2_x-1	107.86	48.13	331.37	150.75	159.14	147.51	3.52	2.31	0.85	50.28	22.71	0.81	1.30	2.65	0.88	0.81	2.24	0.30	0.95	2.21	0.26
P_2_x-2	115.09	48.60	331.44	156.67	98.28	148.59	3.64	1.75	0.85	46.09	16.28	0.70	1.35	2.02	0.88	0.81	2.37	0.34	1.59	2.83	0.26
P_2_x-3	100.37	45.55	337.96	152.27	144.75	134.75	3.59	2.06	0.84	44.76	15.81	0.80	1.31	2.33	0.88	0.72	2.20	0.29	1.05	2.83	0.27
P_2_x-4	111.55	49.78	338.48	159.94	164.01	148.65	3.67	2.42	0.88	48.53	20.36	0.75	1.34	2.75	0.87	0.80	2.24	0.27	0.98	2.38	0.27
P_2_x-5	99.39	47.17	343.08	154.32	87.62	142.60	3.09	1.03	0.84	41.83	13.10	0.67	1.11	1.15	0.86	0.76	2.11	0.29	1.76	3.19	0.24
P_2_x-6	96.82	46.05	343.79	144.12	70.00	128.32	3.07	0.95	0.78	40.15	12.32	0.61	1.12	1.07	0.88	0.69	2.10	0.29	2.06	3.26	0.24
P_2_x-7	98.83	44.14	347.79	146.95	77.46	136.37	3.35	1.67	0.82	40.87	14.23	0.62	1.25	1.92	0.87	0.75	2.24	0.28	1.90	2.87	0.27
P_2_x-8	100.83	45.27	349.89	142.43	83.62	133.91	3.30	2.40	0.82	42.72	14.65	0.60	1.20	2.69	0.87	0.72	2.23	0.27	1.70	2.92	0.26
P_2_x-9	98.33	43.61	350.34	140.84	85.88	126.41	3.36	1.38	0.79	41.94	16.40	0.63	1.24	1.58	0.89	0.69	2.25	0.28	1.64	2.56	0.27
P_2_x-10	105.66	45.54	350.70	151.73	110.38	138.48	3.38	1.68	0.83	46.67	19.02	0.72	1.24	1.92	0.89	0.75	2.32	0.30	1.37	2.45	0.26
P_2_x-11	105.64	47.03	351.54	149.48	152.61	137.61	3.53	1.49	0.82	44.77	14.99	0.83	1.30	1.70	0.88	0.75	2.25	0.32	0.98	2.99	0.27
P_2_x-12	97.86	45.05	353.25	147.60	90.81	138.14	3.26	1.02	0.82	43.23	15.74	0.66	1.20	1.16	0.87	0.75	2.17	0.28	1.63	2.75	0.25
P_2_x-13	92.02	44.32	355.41	132.03	90.70	134.52	3.43	1.05	0.81	44.28	14.82	0.61	1.23	1.16	0.87	0.71	2.08	0.25	1.46	2.99	0.24
P_2_x-14	96.55	43.52	357.79	138.02	148.39	134.39	3.61	3.05	0.78	42.68	14.18	0.78	1.30	3.40	0.86	0.71	2.22	0.28	0.93	3.01	0.28
P_2_x-15	99.03	42.77	364.14	139.45	94.00	136.21	3.54	1.18	0.79	42.33	15.97	0.59	1.31	1.36	0.88	0.74	2.32	0.25	1.48	2.65	0.26
P_2_x-16	102.93	43.07	364.42	141.21	90.22	134.56	3.43	1.80	0.81	42.31	15.29	0.61	1.25	2.03	0.88	0.72	2.39	0.28	1.57	2.77	0.26
P_2_x-17	97.76	44.58	366.54	157.09	95.44	148.11	3.62	1.69	0.91	47.54	15.75	0.70	1.29	1.86	0.86	0.78	2.19	0.27	1.65	3.02	0.26
P_2_x-18	104.74	46.13	369.93	150.34	93.34	141.69	3.56	2.19	0.90	44.57	17.02	0.60	1.32	2.53	0.88	0.78	2.27	0.26	1.61	2.62	0.26
P_2_x-19	99.28	41.95	370.44	161.60	114.19	129.96	3.17	0.85	0.92	40.83	13.43	0.71	1.14	0.95	0.82	0.69	2.37	0.27	1.42	3.04	0.23
P_2_x-20	101.07	32.09	371.85	159.10	147.44	122.84	3.25	0.69	0.88	39.41	14.62	0.80	1.18	0.78	0.82	0.66	3.15	0.29	1.08	2.69	0.25
P_2_x-21	112.69	40.20	385.66	165.69	151.83	144.65	3.39	0.95	0.97	41.06	16.05	0.69	1.22	1.06	0.79	0.77	2.80	0.26	1.09	2.56	0.24
P_2_x-22	112.10	32.82	390.58	163.95	137.56	135.17	3.33	0.84	0.90	39.63	16.60	0.71	1.25	0.97	0.82	0.75	3.42	0.29	1.19	2.39	0.25
P_2_x-23	96.63	33.00	390.84	170.02	109.42	132.02	3.12	0.75	0.88	41.33	13.80	0.70	1.15	0.85	0.81	0.72	2.93	0.25	1.55	2.99	0.24
P_2_x-24	98.66	42.57	393.16	159.37	157.91	129.97	3.15	0.79	0.86	41.38	13.89	0.76	1.14	0.88	0.84	0.70	2.32	0.25	1.01	2.98	0.23
P_2_x-25	109.45	42.62	393.17	151.97	139.27	136.22	3.19	1.13	0.88	43.62	17.24	0.68	1.17	1.29	0.85	0.74	2.57	0.27	1.09	2.53	0.24
P_2_x-26	106.02	43.97	405.03	151.20	153.68	135.98	3.26	1.07	0.82	42.82	14.69	0.77	1.20	1.22	0.87	0.74	2.41	0.29	0.98	2.92	0.25
P_2_x-27	103.82	42.04	406.27	151.38	123.38	140.54	3.25	1.38	0.84	44.12	16.52	0.74	1.19	1.56	0.86	0.76	2.47	0.30	1.23	2.67	0.25
P_2_x-28	109.81	40.67	409.09	136.26	99.59	142.84	3.35	1.10	0.77	44.09	17.09	0.57	1.27	1.28	0.90	0.80	2.70	0.27	1.37	2.58	0.25
P_2_x-29	100.63	41.83	409.74	135.23	112.27	135.09	3.35	1.50	0.77	37.44	12.67	0.69	1.25	1.73	0.89	0.74	2.41	0.30	1.20	2.95	0.26
P_2_x-30	114.97	43.53	428.61	140.04	90.24	138.26	3.43	2.32	0.79	41.79	15.44	0.57	1.28	2.69	0.87	0.76	2.64	0.29	1.55	2.71	0.25

Analytical results of the rare earth elements of 30 mudstones are presented in Table 5. The total rare earth elements (∑REE) ranges from 148.14 μg/g to 184.37 μg/g (avg. of 162.49 μg/g), which is higher than the ∑REE in the Upper Continental Crust (UCC) (146.37 μg/g). The ratio of light rare earth elements to heavy rare earth elements (LREE/HREE) in the samples ranges from 7.02 to 9.59 (avg. of 7.48), indicating a significant enrichment of LREE relative to HREE (Table 5). The (La/Yb)_N_ of the samples ranged from 8.19 to 9.60 (avg. of 8.82), indicating significant fractionation between LREE and HREE. The Chondrite-normalized REE distribution patterns of the Xueyuanhe Formation mudstones are provided in Figure 3. The La–Eu segment of the curve exhibits a steep slope, indicating strong LREE fractionation, whereas the Gd–Lu segment is relatively flat, suggesting weak HREE fractionation. All samples display similar REE distribution patterns, comparable to that of the UCC, suggesting a consistent provenance likely derived from the upper continental crust.^22^ All samples have strong negative Eu anomalies (0.61–0.66, avg. of 0.64) and strong negative Ce anomalies (0.75–0.82, avg. of 0.79) (Table 5).

**Figure 3:**
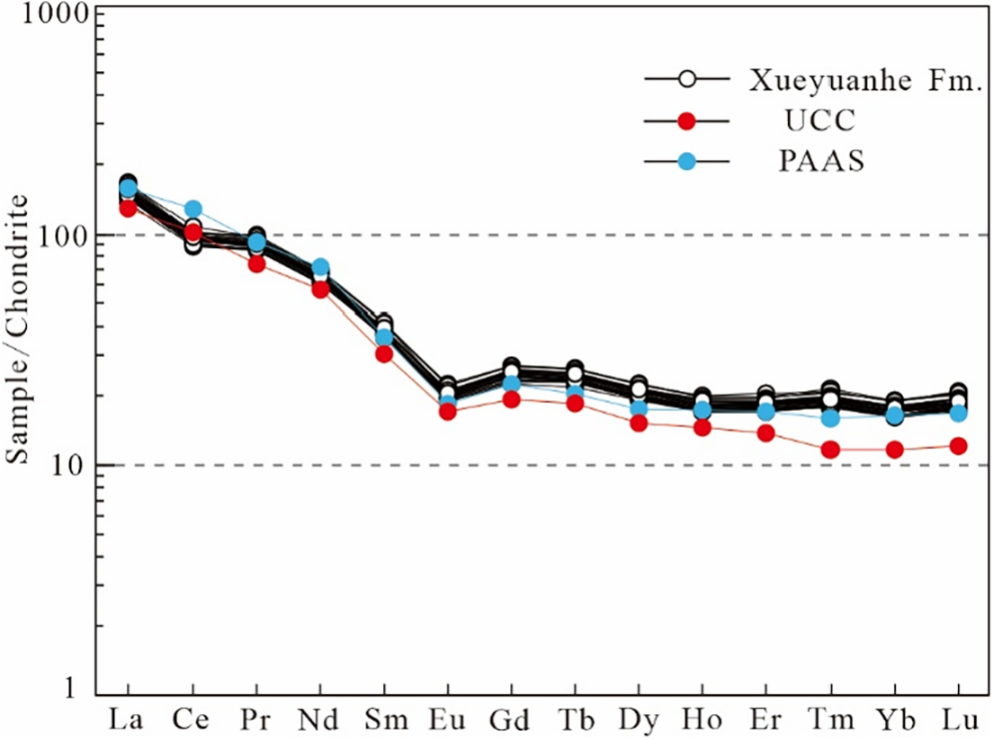
Chondrite-normalized REE patterns of mudstones from the Xueyuanhe Formation in Raggyorcaka Area.

**Table 5:** Rare Earth Element Contents (μg/g) and Geochemical Parameters of Mudstones from the Xueyuanhe Formation in Raggyorcaka Area

Sample	Sc	Y	La	Ce	Pr	Nd	Sm	Eu	Gd	Tb	Dy	Ho
P_2_x-1	16.99	30.34	39.31	61.70	8.91	31.48	6.21	1.24	5.44	0.94	5.49	1.09
P_2_x-2	17.54	31.58	37.72	63.40	9.28	32.46	6.35	1.32	5.62	1.00	5.74	1.13
P_2_x-3	16.66	26.92	37.36	58.18	8.52	29.84	5.87	1.15	5.02	0.86	4.94	0.99
P_2_x-4	17.79	30.90	40.22	62.19	9.52	32.95	6.45	1.29	5.59	0.99	5.80	1.13
P_2_x-5	17.02	28.80	36.40	59.53	8.55	30.05	5.99	1.21	5.22	0.92	5.33	1.04
P_2_x-6	16.93	27.33	34.49	57.82	8.54	30.12	5.80	1.17	5.08	0.89	5.09	1.02
P_2_x-7	15.88	28.07	34.90	56.32	8.28	29.11	5.73	1.19	5.10	0.90	5.20	0.99
P_2_x-8	16.62	27.78	36.88	58.26	8.49	30.08	5.79	1.15	5.04	0.89	5.04	1.02
P_2_x-9	15.60	26.93	32.75	54.65	8.17	29.10	5.53	1.12	4.80	0.87	4.98	0.98
P_2_x-10	16.72	28.72	37.95	61.45	8.96	31.37	6.02	1.18	5.16	0.90	5.14	1.04
P_2_x-11	16.85	28.27	37.00	58.64	8.82	30.97	6.00	1.20	5.21	0.91	5.29	1.03
P_2_x-12	16.27	29.11	36.75	60.23	8.84	31.21	6.00	1.21	5.27	0.91	5.30	1.04
P_2_x-13	16.36	27.77	39.52	60.70	9.19	31.37	5.91	1.14	5.12	0.88	5.10	1.01
P_2_x-14	15.53	28.79	36.86	61.87	8.62	30.46	6.02	1.21	5.30	0.94	5.39	1.04
P_2_x-15	15.78	29.35	37.01	58.75	8.64	30.51	5.97	1.23	5.36	0.94	5.40	1.06
P_2_x-16	15.74	28.69	35.50	58.62	8.67	30.92	5.98	1.20	5.15	0.93	5.29	1.05
P_2_x-17	17.83	31.50	39.74	66.63	9.32	32.83	6.31	1.29	5.57	0.98	5.74	1.14
P_2_x-18	17.19	30.76	38.71	62.50	9.31	32.40	6.38	1.20	5.42	0.96	5.48	1.11
P_2_x-19	18.34	25.87	35.25	57.51	8.58	30.06	5.66	1.10	4.67	0.82	4.90	0.97
P_2_x-20	16.59	27.05	36.28	56.47	8.26	29.49	5.95	1.21	5.25	0.92	5.21	1.01
P_2_x-21	17.61	27.71	36.89	60.96	8.79	29.54	5.67	1.12	4.77	0.87	5.07	1.03
P_2_x-22	16.58	27.20	35.65	57.77	8.65	30.01	5.68	1.13	4.87	0.87	4.97	1.00
P_2_x-23	16.74	27.52	35.02	57.60	8.48	29.83	5.78	1.17	5.03	0.87	5.00	1.00
P_2_x-24	17.69	26.39	35.45	59.05	8.59	29.84	5.63	1.14	4.85	0.86	4.96	0.98
P_2_x-25	16.79	29.23	34.51	58.90	8.65	31.06	6.02	1.21	5.25	0.91	5.45	1.08
P_2_x-26	16.82	29.77	35.33	59.13	8.76	30.66	6.04	1.21	5.33	0.95	5.48	1.08
P_2_x-27	16.54	29.18	36.47	58.72	8.79	30.73	6.07	1.24	5.37	0.95	5.53	1.09
P_2_x-28	15.46	28.98	37.84	61.30	9.04	31.91	6.08	1.20	5.26	0.92	5.43	1.06
P_2_x-29	15.43	27.16	35.49	55.77	8.32	29.98	5.75	1.15	4.98	0.88	5.17	1.01
P_2_x-30	16.77	29.29	37.58	60.15	8.72	31.03	6.09	1.20	5.28	0.94	5.48	1.09

Sample	Er	Tm	Yb	Lu	Th	∑REE	LREE	HREE	$\frac{\mathrm{LREE}}{\mathrm{HREE}}$	La/Yb	δEu	δCe
P_2_x-1	3.19	0.51	3.10	0.50	13.48	0.81	–8.37	1.30	2.65	9.10	0.64	0.78
P_2_x-2	3.29	0.53	3.28	0.53	13.93	0.70	–7.35	1.35	2.02	8.25	0.66	0.81
P_2_x-3	2.94	0.48	2.83	0.44	13.24	0.80	–7.50	1.31	2.33	9.45	0.63	0.77
P_2_x-4	3.31	0.55	3.26	0.52	13.55	0.75	–5.54	1.34	2.75	8.85	0.64	0.75
P_2_x-5	3.09	0.49	2.92	0.47	13.13	0.67	–6.20	1.11	1.15	8.94	0.65	0.80
P_2_x-6	2.90	0.47	2.80	0.45	12.98	0.61	–1.31	1.12	1.07	8.83	0.64	0.80
P_2_x-7	2.90	0.47	2.80	0.45	12.23	0.62	9.31	1.25	1.92	8.93	0.66	0.79
P_2_x-8	3.04	0.48	2.91	0.46	12.70	0.60	2.71	1.20	2.69	9.08	0.63	0.78
P_2_x-9	2.87	0.47	2.78	0.47	12.33	0.63	9.83	1.24	1.58	8.44	0.65	0.80
P_2_x-10	3.13	0.50	3.02	0.47	13.17	0.72	8.23	1.24	1.92	9.02	0.63	0.79
P_2_x-11	3.05	0.48	2.95	0.47	13.03	0.83	11.23	1.30	1.70	8.98	0.64	0.77
P_2_x-12	3.14	0.51	3.02	0.48	12.83	0.66	11.44	1.20	1.16	8.72	0.65	0.79
P_2_x-13	3.01	0.49	2.95	0.48	14.09	0.61	3.31	1.23	1.16	9.60	0.62	0.75
P_2_x-14	3.05	0.49	2.96	0.48	12.90	0.78	8.03	1.30	3.40	8.92	0.64	0.82
P_2_x-15	3.11	0.50	3.05	0.49	13.43	0.59	24.16	1.31	1.36	8.71	0.65	0.78
P_2_x-16	3.12	0.50	3.06	0.48	12.96	0.61	18.40	1.25	2.03	8.32	0.65	0.79
P_2_x-17	3.43	0.53	3.24	0.54	13.82	0.70	13.06	1.29	1.86	8.79	0.65	0.82
P_2_x-18	3.26	0.54	3.24	0.53	13.71	0.60	32.11	1.32	2.53	8.57	0.61	0.78
P_2_x-19	2.85	0.46	2.83	0.45	13.73	0.71	19.19	1.14	0.95	8.93	0.64	0.79
P_2_x-20	2.91	0.46	2.76	0.44	13.18	0.80	26.34	1.18	0.78	9.42	0.65	0.77
P_2_x-21	3.08	0.49	2.97	0.48	13.97	0.69	35.96	1.22	1.06	8.90	0.64	0.80
P_2_x-22	3.00	0.49	2.89	0.48	13.48	0.71	53.94	1.25	0.97	8.84	0.64	0.78
P_2_x-23	2.92	0.48	2.82	0.46	12.86	0.70	48.05	1.15	0.85	8.90	0.65	0.79
P_2_x-24	2.94	0.47	2.90	0.46	13.79	0.76	45.77	1.14	0.88	8.77	0.65	0.80
P_2_x-25	3.16	0.50	3.02	0.50	13.12	0.68	50.30	1.17	1.29	8.19	0.64	0.81
P_2_x-26	3.17	0.51	3.09	0.50	13.24	0.77	62.76	1.20	1.22	8.21	0.64	0.80
P_2_x-27	3.18	0.51	3.11	0.50	13.24	0.74	61.65	1.19	1.56	8.41	0.65	0.78
P_2_x-28	3.14	0.51	3.06	0.50	13.36	0.57	75.93	1.27	1.28	8.88	0.63	0.79
P_2_x-29	2.97	0.48	2.90	0.47	13.01	0.69	71.92	1.25	1.73	8.77	0.64	0.77
P_2_x-30	3.13	0.50	3.03	0.48	13.46	0.57	92.18	1.28	2.69	8.89	0.63	0.79

## 5 Discussion

### 5.1 Source Rock Evaluation

#### 5.1.1 Organic Matter Abundance

The organic matter abundance refers to the percentage of organic matter in the source rock and is generally considered to be positively correlated with hydrocarbon generation potential. The indicators for evaluating the organic matter abundance of source rocks include TOC, chloroform bitumen “A”, hydrocarbon generation potential (*S*_1_ + *S*_2_), total hydrocarbon content (HC), and hydrogen index (HI).^23^ The samples studied are highly mature to overmature, present-day TOC, *S*_1_, *S*_2_ and HI values may underestimate the original hydrocarbon generation potential. Therefore, the evaluation in this study mainly reflects the residual source rock quality. The Chinese Petroleum Industry Standard (SY/T5735–2019) classifies the organic matter abundance grades of carbonate rocks and mudstones into four categories: nonsource rocks (TOC < 0.5%, “A” < 0.05%), general source rocks (0.5% < TOC < 1%, 0.05% < “A” < 0.1%), good source rocks (1% < TOC < 2%, 0.1% < “A” < 0.2%), and high-quality source rocks (TOC > 2%, “A” ≥ 0.2%). The TOC content of 30 mudstone samples from the Xueyuanhe Formation ranged from 0.42% to 0.60% (avg. of 0.52%) (Table 1). Among them, 5 samples had TOC content lower than 0.5%, which belonged to nonhydrocarbon source rocks. Twenty-five samples had TOC content ranging from 0.5% to 0.6%, which belonged to general hydrocarbon source rocks. The chloroform bitumen “A”, *S*_1_, *S*_2_, and *S*_1_ + *S*_2_ contents of 20 mudstone samples tested were 0.006–0.014% (avg. of 0.009%), 0.01–0.02 mg/g (avg. of 0.02 mg/g), 0.08–0.12 mg/g (avg. of 0.1 mg/g), and 0.09–0.14 mg/g (avg. of 0.12 mg/g), respectively (Table 1). All these values fall within the nonsource rocks range. Based on the analysis of TOC, chloroform bitumen “A”, and the hydrocarbon generation potential, it is concluded that the Xueyuanhe Formation mudstones in the Raggyorcaka area exhibit poor hydrocarbon generation potential and are overall classified as poor source rocks (Figure 4).

**Figure 4:**
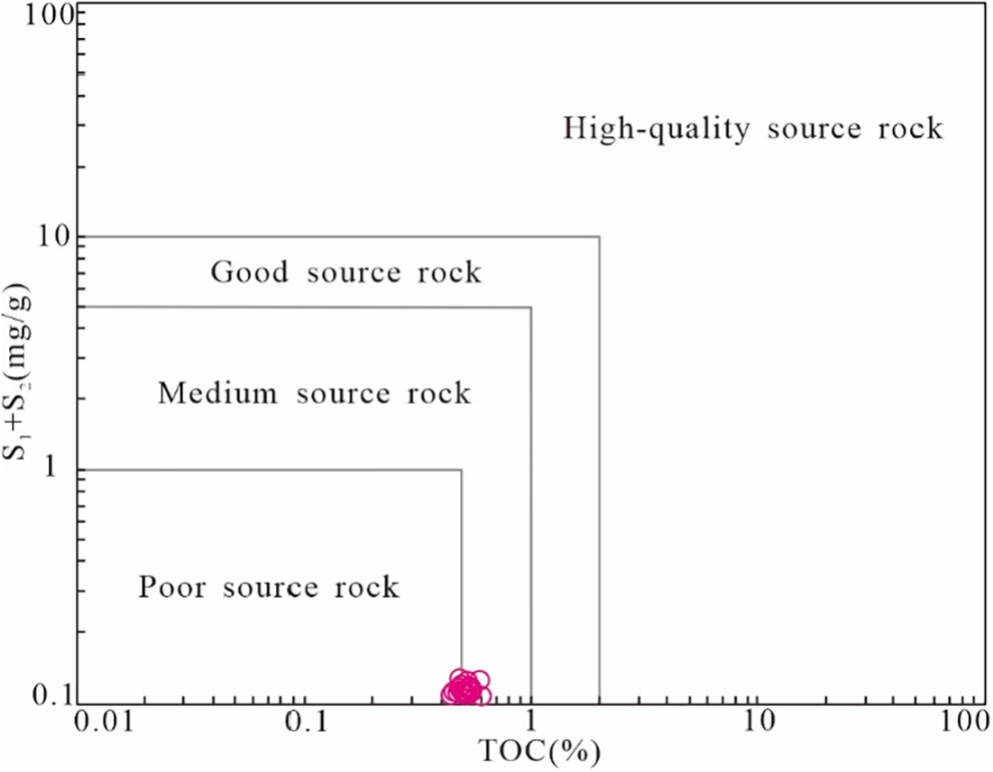
Discrimination diagram of the organic matter abundance of mudstones from the Xueyuanhe Formation in Raggyorcaka area.

#### 5.1.2 Organic Matter Type

The sources and preservation conditions of initial organic matter can vary considerably, and consequently, source rocks may show significant differences in their hydrocarbon generation potential. Therefore, to objectively assess the hydrocarbon generation potential of source rocks, it is necessary to evaluate the types of organic matter.^24^ Commonly used indicators for evaluating organic matter types include kerogen maceral composition, kerogen elemental composition, carbon isotope composition, and Rock-Eval pyrolysis parameters. Maceral composition analysis reveals that exinite is the dominant component in the Xueyuanhe Formation mudstones, with subordinate vitrinite and minor inertinite. Among the 10 mudstone samples, the kerogen index (TI) of 9 samples ranged from 0.5 to 15.5 (avg. of 7.0), belonging to type II_2_ kerogen; the TI value of 1 sample (P_2_x-25) was −9.3, belonging to type III kerogen (Table 2).

The elemental composition of kerogen is closely related to the organic matter type, and it can be determined by the H/C and O/C atomic ratio. The H/C atomic ratio range of 10 mudstone samples in the study area was 0.05–0.06 (avg. of 0.05), and the O/C atomic ratio range was 0.07–0.09 (avg. of 0.08). All samples fall within the lower left field of the H/C–O/C diagram, indicating a high degree of mature to overmature evolution. Using this method, distinguishing kerogen types is difficult, as increasing maturity causes the H/C and O/C ratios to decrease and converge,^12^ consistent with the later evaluation of organic matter maturity.

Based on integrated analyses of kerogen maceral and elemental compositions, the organic matter type of the Xueyuanhe Formation mudstones is characterized as predominantly type II_2_ kerogen, with minor type III kerogen, indicating generally good organic matter quality.

#### 5.1.3 Organic Matter Maturity

Organic matter maturity is an important parameter for determining whether source rocks are effective. Commonly used indicators for evaluating maturity include vitrinite reflectance (Ro), kerogen color and fluorescence, Rock-Eval pyrolysis parameters, and biomarker distributions.^25^ Among them, Ro is the most effective and widely used indicator for evaluating the maturity of source rocks and has been extensively applied to assess the thermal maturity of post-Permian sediments. Generally, Ro values increase progressively with increasing thermal maturity. Ro values less than 0.5% correspond to immature stage, 0.5%–0.7% correspond to low-mature stage, 0.7%–1.3% correspond to mature stage, 1.3%–2% correspond to highly mature stage, and Ro values greater than 2% represent overmature stage. As thermal maturity advances, the organic matter undergoes a progressive transformation, generating oil at lower maturity stage, then wet gas, and finally dry gas at the highest maturity stages.^26^ The Ro values of 10 samples from the study area range from 0.85% to 1.94% (avg. of 1.72%), indicating that the organic matter has reached the high maturity stage. Furthermore, during the burial process of organic matter, as maturity increases, the activation energy required for converting residual organic matter into hydrocarbons also increases. Consequently, the temperature required to generate hydrocarbons gradually rises. Thus, the maximum pyrolysis temperature (*T*_max_) is also a reference indicator for determining organic matter maturity.^27^ The *T*_max_ values of 20 samples from the study area ranged from 524 to 557 °C (avg. of 554 °C), indicating that the organic matter has entered the overmature stage, which is consistent with the Ro-*T*_max_ maturity discrimination diagram (Figure 5). Overall, the organic matter of the Xueyuanhe Formation mudstones exhibits a high degree of thermal evolution, placing it predominantly in the high- to overmaturity stage.

**Figure 5:**
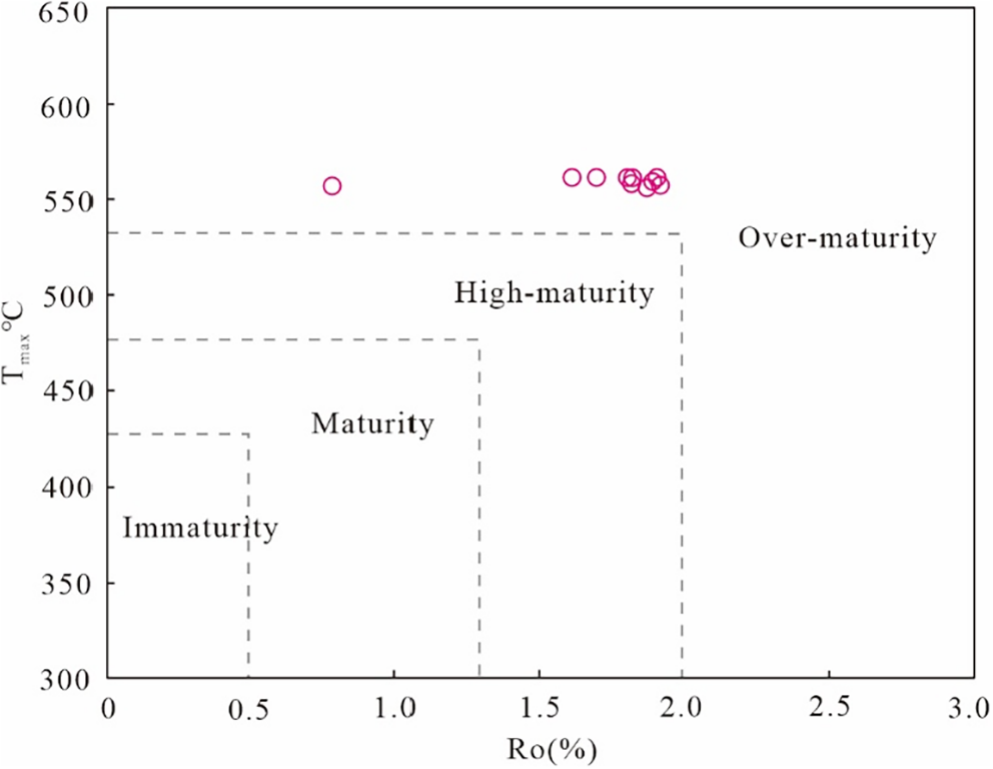
Discrimination diagram of the abundance of organic matter of mudstones from the Xueyuanhe Formation in Raggyorcaka area

In summary, although the mudstones of the Xueyuanhe Formation are favorable for gas generation (dominated by type II_2_ kerogen), their low organic matter abundance and high degree of thermal evolution indicate poor overall hydrocarbon generation potential.

### 5.2 Paleoenvironmental Conditions

#### 5.2.1 Paleoclimate and Terrigenous Detritus Input

Paleoclimate conditions control the process of sedimentary basin receiving terrigenous detritus, thereby influencing the enrichment and preservation of organic matter in the sediments. Since elemental proxies may be influenced by provenance, grain size, detrital input, and diagenetic modification, no single proxy is used independently in this study. Paleoenvironmental interpretations are based on the consistency of multiple geochemical indicators. Various geochemical indicators, such as the Chemical Index of Alteration (CIA), *C*-value, and Sr/Cu ratio, are widely applied in paleoclimate studies.^28^

The CIA is calculated as follows CIA = [(Al_2_O_3_)/(Al_2_O_3_ + CaO* + Na_2_O + K_2_O)] × 100, where CaO* only represents the content of CaO in silicate minerals. In this study, the obtained P_2_O_5_ data were used for preliminary calibration of CaO according to (CaO* = CaO – P_2_O_5_ × 10/3).^29^ When the residual molar content is less than Na_2_O, CaO value is used as CaO* value; otherwise, Na_2_O value is used as CaO* value.^30,31^ CIA values between 50 and 60 indicate a dry and cold climate with weak chemical weathering; values between 60 and 80 suggest a warm and humid climate with moderate chemical weathering; and values between 80 and 100 reflect a hot and humid climate with intense chemical weathering.^32,33^ The CIA values of the mudstone samples in the study area range from 78.38 to 79.29 (avg. of 78.82) (Figure 6), indicating moderate chemical weathering during the depositional period.

**Figure 6:**
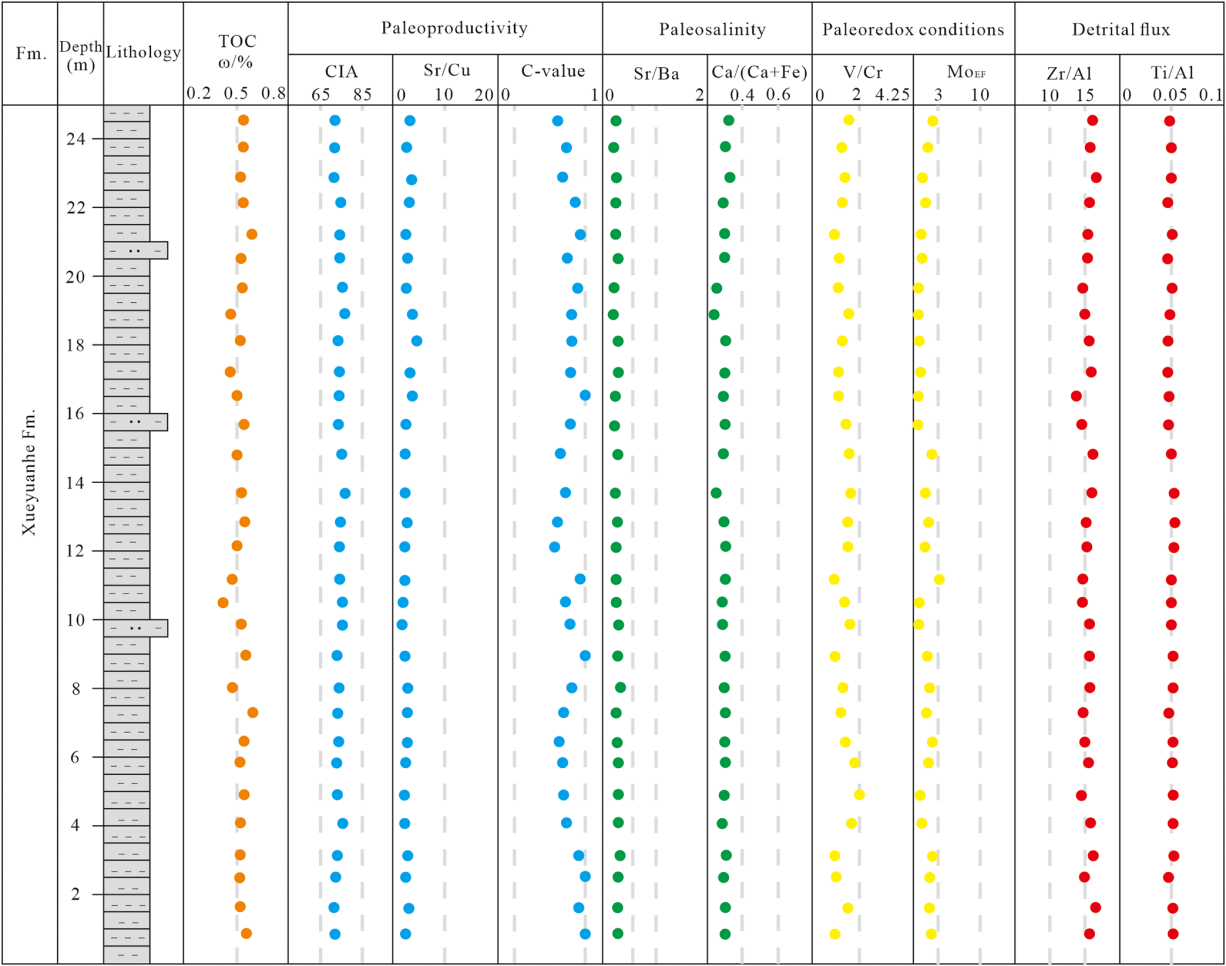
Paleoenvironmental parameters of mudstones from the Xueyuanhe Formation in Raggyorcaka Area.

Humid climatic conditions favor the relative enrichment of elements such as Mn, Fe, Cr, Ni, V, and Co in sediments. In arid environments, however, evaporation promotes extensive precipitation of elements like Mg, Ca, Na, K, Ba, and Sr, leading to their relative enrichment through the formation of saline deposits. Therefore, the ratio of these two types of elements (the *C*-value) can be used to reflect the paleoclimate changes during the sedimentation period. The *C*-value is calculated as follows *C* = ∑(Fe + Mn + Cr + Ni + V + Co)/∑(Ca + Mg + Sr + Ba + K + Na).^34^ Previous studies have shown that *C* < 0.2, 0.2 < *C* < 0.8, *C* > 0.8 indicate arid, semiarid to semihumid, and humid paleoclimate conditions, respectively.^35^ The *C*-values of the mudstone samples range between 0.57 and 0.83 (avg. of 0.69) (Figure 6), with the exception for two samples exceeding 0.8, these values suggest that the study area experienced predominantly semiarid to semihumid climatic conditions during deposition. In addition, the Sr/Cu ratio in mudstones responds sensitively to temperature variations, increasing progressively with the degree of aridification. Thus, it serves as a valuable indicator for reconstructing paleoclimatic conditions.^36,37^ Generally, Sr/Cu > 10 indicates a hot and arid environment, whereas 1 < Sr/Cu < 10 represents a warm and humid sedimentary environment.^38^ The Sr/Cu ratio of the mudstone samples in the study area is relatively low and stable, ranging from 2.08 to 3.42 (avg. of 2.41) (Figure 6), indicating a relatively warm and humid climate condition during the depositional period.

The input of terrigenous detritus can have a certain diluting effect on the enrichment process of the original organic matter.^39^ Elements such as Al, Ti, and Zr, due to their relatively stable chemical properties, are less affected by weathering and diagenesis, and thus are important indicators for evaluating the intensity of terrigenous detritus input. Among them, Al mainly originates from the aluminum-silicate clay minerals in the terrigenous detritus. It is usually regarded as an evaluation indicator for fine particles input. Meanwhile, Ti and Zr elements not only come from clay minerals but also from coarser-grained minerals. Therefore, the enrichment factor (EF) of Ti and Zr elements and their ratios to Al can effectively reflect the input degree of coarse-grained debris.^40^ The Al, Ti, and Zr contents of the Xueyuanhe Formation mudstones in the study area were 8.54 wt %–9.06 wt % (avg. of 8.81 wt %), 0.42 wt %–0.47 wt % (avg. of 0.45 wt %), and 122.84 μg/g–148.65 μg/g (avg. of 137.14 μg/g), respectively. The Ti_EF_ and Zr_EF_ were 0.79–0.90 (avg. of 0.86) and 0.66–0.81 (avg. of 0.74), respectively. All samples showed relatively weak depletion, indicating limited input of coarse-grained terrigenous detritus during the depositional period of the Xueyuanhe Formation in this study area. In addition, the relatively low and stable Zr/Al and Ti/Al ratios (13.87–17.10 and 0.047–0.053, respectively) also indicate that there was less input of terrigenous detritus during sedimentation period (Figure 6). However, continuous fine-grained felsic aluminosilicate input may still exist, as reflected by the high Al_2_O_3_ and clay-rich mudstone composition. This fine-grained detrital supply may have diluted organic matter without indicating strong high-energy clastic influx.

The sedimentation rate also significantly affects the organic matter content in the unit thickness of the deposited strata. The (La/Yb)_N_ value of rare earth elements can be effectively used to characterize the sedimentation rate.^41^ When the degree of rare earth element fractionation is strong, the (La/Yb)_N_ value of the sample will be significantly greater than or less than 1, indicating a slow sedimentation rate; conversely, when the degree of rare earth element fractionation is weak, the (La/Yb)_N_ value will be close to 1.0, indicating a rapid sedimentation rate.^41^ The (La/Yb)_N_ value of the samples in this study ranged from 8.19 to 9.60 (avg. of 8.82), indicating that the sedimentation rate of the Xueyuanhe Formation mudstones was slow, which further inhibited the enrichment process of organic matter.

#### 5.2.2 Redox Conditions

The redox conditions of water bodies can directly affect the preservation of organic matter.^42,43^ The trace elements such as V, U, Mo, Ni, Co, and Cr in sediments are sensitive to the redox environment of water bodies. They have the characteristics of being prone to migration in an oxidizing environment and precipitation in a reducing environment. The ratio of their contents and the derived enrichment factor are commonly used identification indicators for restoring the redox environment of water bodies. It is generally believed that when V/Cr < 2, Ni/Co < 5, and U/Th < 0.75, it indicates an oxic environment; when 2 < V/Cr < 4.25, 5 < Ni/Co < 7, and 0.75 < U/Th < 1.25, it indicates a suboxic environment; and when V/Cr > 4.25, Ni/Co > 7, and U/Th > 1.25, it represents an anoxic environment.^44^ The V/Cr, Ni/Co, and U/Th ratios of the mudstone samples from the Xueyuanhe Formation range from 0.93–2.06 (avg. of 1.37), 2.21–3.26 (avg. of 2.78), and 0.23–0.28 (avg. of 0.25), respectively. These element ratios all fall within the range of oxidized environment (Figure 5), indicating an oxidized water body environment overall (Figure 6).

The sediments exhibit strong inheritance of rare earth elements from their source rocks. The distribution characteristics of Eu and Ce can effectively reflect the redox conditions of the sedimentary water bodies.^45^ Ce has two valence states, Ce^3+^ and Ce^4+^. Under oxidative conditions, Ce^3+^ readily oxidizes to Ce^4+^ and is adsorbed by iron–manganese oxide colloids, resulting in precipitation and a decrease in the concentration of Ce in seawater. Under reducing conditions, the iron–manganese oxide colloids dissolve, and Ce^4+^ is reduced to Ce^3+^. At this time, the Ce in the water body will exhibit a relatively weak negative anomaly or even a local positive anomaly.^46,47^ Eu has two valence states, Eu^2+^ and Eu^3+^. In modern seawater, the Eu element exists in the form of Eu^3+^. However, in a strongly reducing environment, Eu^3+^ can be reduced to Eu^2+^ and enter the carbonate rock lattice, resulting in a positive anomaly of Eu in the water body. Studies have shown that when the values of δEu and δCe are greater than 1, it indicates a reducing environment; conversely, the smaller the values of δEu and δCe, the shallower and more oxic the sedimentary water body is.^48^ The δEu values of the mudstone samples in the study area range from 0.61 to 0.66 (avg. of 0.64), and the δCe values range from 0.75 to 0.82 (avg. of 0.79). Both show strong negative anomalies, indicating the oxic characteristics of the sedimentary paleoenvironment.

The Mo_EF_–U_EF_ covariant diagram can be used to determine the degree of water body limitation and the redox conditions.^49^ The Mo enrichment factor (Mo_EF_) of the mudstone samples from the Xueyuanhe Formation ranged from 0.78 to 3.40, while the U enrichment factor (U_EF_) ranged from 1.11 to 1.35. Among the 30 samples, only five samples had a Mo_EF_ value slightly less than 1, while the remaining 25 samples all had Mo_EF_ values greater than 1, showing an overall enrichment feature; The U_EF_ values, on the other hand, showed relatively small variations and exhibited a weak enrichment feature. In the Mo_EF_–U_EF_ covariant pattern diagram (Figure 7), all samples fall within the oxic environment area, indicating an unrestricted marine environment, which is consistent with the previous results indicated by the values of V/Cr, Ni/Co, U/Th, δEu, and δCe.

**Figure 7:**
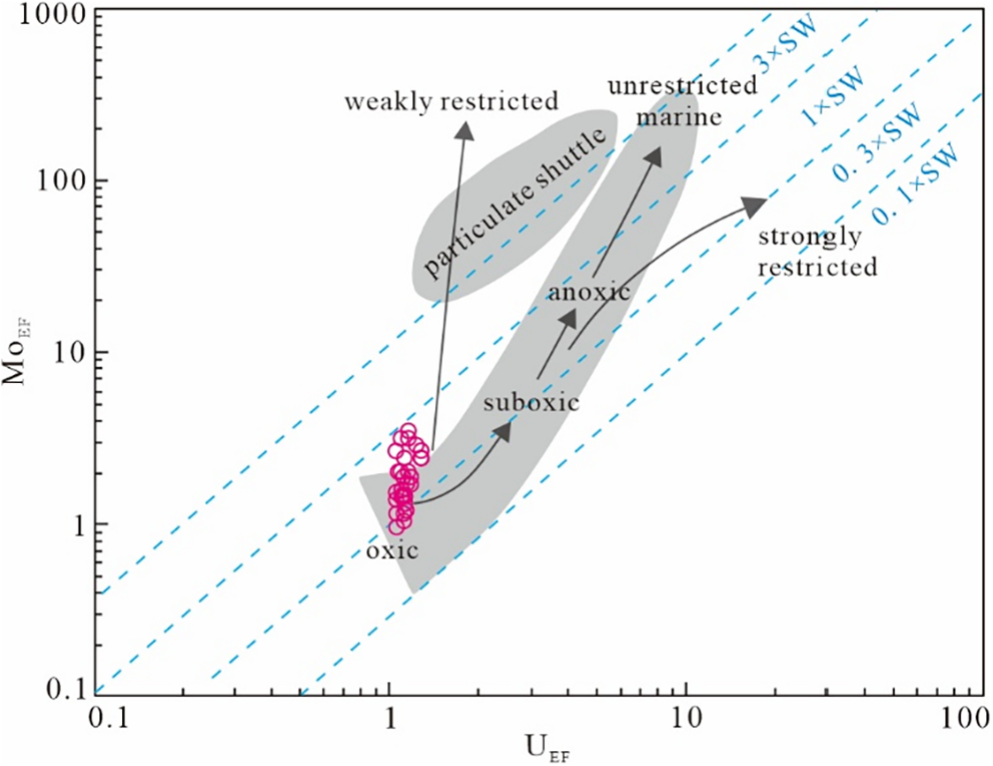
Mo_EF_–U_EF_ covariant pattern diagram of mudstones from the Xueyuanhe Formation in Raggyorcaka area.

#### 5.2.3 Paleosalinity and Paleo-Water-Depth

The Sr/Ba and Ca/(Ca + Fe) ratios have a significant correlation with the salinity of the sedimentary water body, and they are commonly used indicators for determining the paleosalinity and the corresponding climatic conditions.^50,51^ Studies have shown that when Sr/Ba < 0.2 and Ca/(Ca + Fe) < 0.4, it represents a freshwater environment; when 0.2 < Sr/Ba < 0.5 and 0.4 < Ca/(Ca + Fe) < 0.6, it represents a brackish water environment; and when Sr/Ba > 0.5 and Ca/(Ca + Fe) > 0.6, it represents a saltwater environment.^51^ The data analysis results of this study show that the Sr/Ba and Ca/(Ca + Fe) ratios of the Xueyuanhe Formation mudstones are relatively stable, with ratio ranges from 0.25 to 0.34 (avg. of 0.28), and the Ca/(Ca + Fe) ratio ranges from 0.19 to 0.33 (avg. of 0.28), indicating that the sedimentary period of the Xueyuanhe Formation mudstones in the study area was a low-salinity freshwater environment, corresponding to a warm and humid ancient climate (Figure 6).

The content of trace elements in source rocks also provides important constraints on the reconstruction of the depositional environment. When Mo > 5 × 10^–6^, Co > 40 × 10^–6^, Cu > 90 × 10^–6^, Ba > 1000 × 10^–6^, Ce > 100 × 10^–6^, Pr > 10 × 10^–6^, Nd > 50 × 10^–6^, Sm > 15 × 10^–6^, Gd > 15 × 10^–6^, and U < 1 × 10^–6^, Sn < 3 × 10^–6^, the possibility of the rock layer forming in a water environment above 250 m is relatively high. Conversely, deviation from these thresholds indicates shallow-water deposition.^52^ The Mo content of the Xueyuanhe Formation mudstones ranges from 0.69 × 10^–6^ to 3.05 × 10^–6,^ the Co content ranges from 12.32 × 10^–6^ to 22.71 × 10^–6^, the Cu content ranges from 32.09 × 10^–6^ to 49.78 × 10^–6^, the Ba content ranges from 331.37 × 10^–6^ to 428.61 × 10^–6^, the Ce content ranges from 54.65 × 10^–6^ to 66.63 × 10^–6^, the Pr content ranges from 8.17 × 10^–6^ to 9.52 × 10^–6^, the Nd content ranges from 29.10 × 10^–6^ to 32.95 × 10^–6^, the Sm content ranges from 5.53 × 10^–6^ to 6.45 × 10^–6^, the Gd content ranges from 4.67 × 10^–6^ to 5.62 × 10^–6^, the U content ranges from 3.07 × 10^–6^ to 3.67 × 10^–6^, and the Sn content ranges from 2.11 × 10^–6^ to 2.58 × 10^–6^. The contents of all these elements indicate that the Xueyuanhe Formation mudstones were formed in a shallow water environment with a depth of less than 250 m. Furthermore, the Mn element, due to its stable geochemical properties, can be transported over long distances and accumulate in deep water environments far from the shore. Therefore, its content can also serve as a reference indicator for determining the depth of sedimentary water body and the distance from the shore.^53,54^ The Mn element content of the mudstone samples in the study area ranged from 448.50 to 1193.41 μg/g (avg. of 726.09 μg/g). Except for 6 samples, the Mn content of the remaining 24 samples was lower than that of the Archean shale in Australia.^22^ This reflects that the water body was relatively shallow during the sedimentation of the Xueyuanhe Formation mudstones, which is consistent with the oxidized environment determined in the previous analysis.

#### 5.2.4 Hydrothermal Activity and Primary Productivity

Hydrothermal processes can provide the necessary nutrients and energy for life activities and promote the chemical cycling within the Earth’s interior as well as the formation of mineral resources.^55,56^ Studies have shown that the enrichment of oxides such as Fe and Mn is usually associated with hydrothermal processes, and their content ratios and discrimination diagrams are effective indicators for determining whether hydrothermal processes were involved in the sedimentation process.^57,58^ When Fe/Ti > 20, (Fe + Mn)/Ti > 20 ± 5, and Al/(Al + Fe + Mn) < 0.35, it is considered that the sediment has been affected by hydrothermal fluids.^59,60^ The Fe/Ti, (Fe + Mn)/Ti, and Al/(Al + Fe + Mn) ratios of the mudstone samples in the study area range from 9.11 to 10.61 (avg. of 9.78), 9.24 to 10.79 (avg. of 9.94), and 0.65–0.68 (avg. of 0.66), respectively. This indicates that there was no hydrothermal activity during the sedimentary period. Moreover, in the Fe/Ti–Al/(Al + Fe + Mn) and Al–Fe–Mn discrimination diagrams (Figure 8a,b), all samples fall into the Nonhydrothermal area, indicating that the Xueshenguan Formation mudstone was not affected by hydrothermal activity during the sedimentary period and was normal seawater sedimentation.

**Figure 8:**
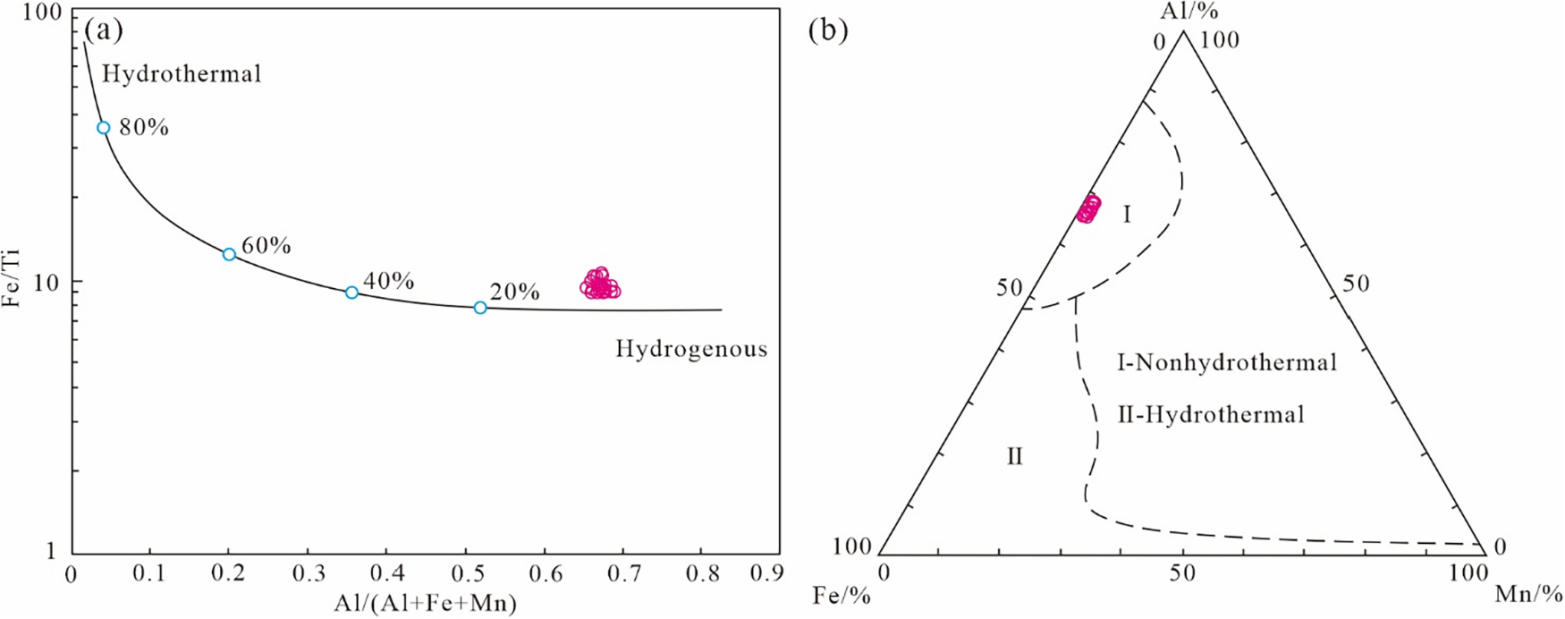
Discrimination diagram of the hydrothermal activity of mudstones from the Middle Permian Xueyuanhe Formation in Raggyorcaka area. (a) Fe/Ti–Al/(Al + Fe + Mn) diagram; (b) Al–Fe–Mn diagram.

The primary producers in the ocean can control the accumulation of original organic matter through their life activities, thereby indirectly influencing the enrichment of organic matter in the sediments. Elements such as P, Ba, and Si in mudstone are effective indicators for evaluating the primary productivity level.^61^ Among them, P is a necessary nutrient element for the growth of plankton. During the decomposition of organisms, it is preserved in the sediment as autogenic phosphorus minerals. The phosphorus in the sediment comes from two major sources: organic phosphorus and detrital phosphorus.^62,63^ In an oxidizing environment, the element Ba is mainly preserved in the form of Barite (BaSO_4_), and its deposition rate is significantly positively correlated with the primary productivity. The biogenic barium (Ba_bio_) content calculated based on the Ba element content can effectively characterize the primary productivity level under oxidative conditions.^64^ The Si_bio_ content can be calculated as follows Si_bio_ = Si_sample_ – [Al_sample_ × (Si/Al)_PAAS_], where (Si/Al)_PAAS_ of 3.11 was adopted as the background value of average shales.^65^ Biogenic silica (Si_bio_), as the main component of the planktonic organism’s skeleton, has a positive correlation with the primary productivity level and is also a reliable indicator for evaluating primary productivity.^66^ The Ba_bio_ content can be calculated as follows Ba_bio_ = Ba_sample_ – Al_sample_ × (Ba/Al)_detrital_, The (Ba/Al)_detrital_ ratios of crustal rocks range from 0.005 to 0.01, while more recent studies suggested a range of 0.0032–0.0046.^67–69^ In this study, the ratio of 0.0039 was adopted as the detrital and biogenic barium content.

In this study, there was no positive correlation between the P content and the Al_2_O_3_ content in the Xueyuanhe Formation mudstones (*R* ^2^ = 0.2078, *n* = 30), indicating that the P in the samples originated from organic materials, it could truly and effectively reflect the primary productivity level. Meanwhile, the P/Ti ratios can further eliminate the influence of terrigenous detritus input and accurately characterize the nutrient status of the ancient sedimentary environment. That is, the larger the P/Ti value, the higher the productivity. The P/Ti value of the Xueyuanhe Formation mudstones is low, ranging from 0.14 to 0.18 (avg. of 0.16). This reflects the relatively low primary productivity level during the deposition period. Furthermore, the calculated Ba_bio_ and Si_bio_ values range from −8.37 to 92.18 (avg. of 25.32) and −0.44 to 1.48 (avg. of 0.63), respectively. The relatively low contents of Ba_bio_ and Si_bio_ are also consistent with the results reflected by P/Ti ratios, indicating that the primary productivity during deposition of the Xueyuanhe Formation mudstones was relatively low (Figure 6).

### 5.3 Sediment Provenance and Tectonic Setting

#### 5.3.1 Sediment Provenance

Weathering products of source rocks constitute the primary source of detrital material in sediments. Since weathering products from different source rock types exhibit significant differences, the source rock is an important factor influencing the geochemical characteristics of sedimentary rocks. Based on the geochemical parameters of the mudstone samples and a series of discriminant diagrams, this paper conducts a comprehensive analysis of the rock compositions of the sedimentary basin’s source area.^70–72^ In the ∑REE–La/Yb diagram (Figure 9a), sample data are distributed in the intersection area of sedimentary rocks, alkaline basalt, and granite, indicating that the source composition of the mudstone in the study area may have a mixed origin characteristic. In the Hf–La/Th diagram (Figure 9b), all the samples from the study area fall within the felsic island arc source area; in the La/Sc–Co/Th and Al_2_O_3_/TiO_2_–SiO_2_ diagrams (Figure 9c,d), the samples all fall within the felsic volcanic rock area. Overall, it is concluded that the source area of the Permian Xueyuanhe Formation is mainly felsic volcanic rocks, with a small amount of basalt and sedimentary rocks possibly mixed in.

**Figure 9:**
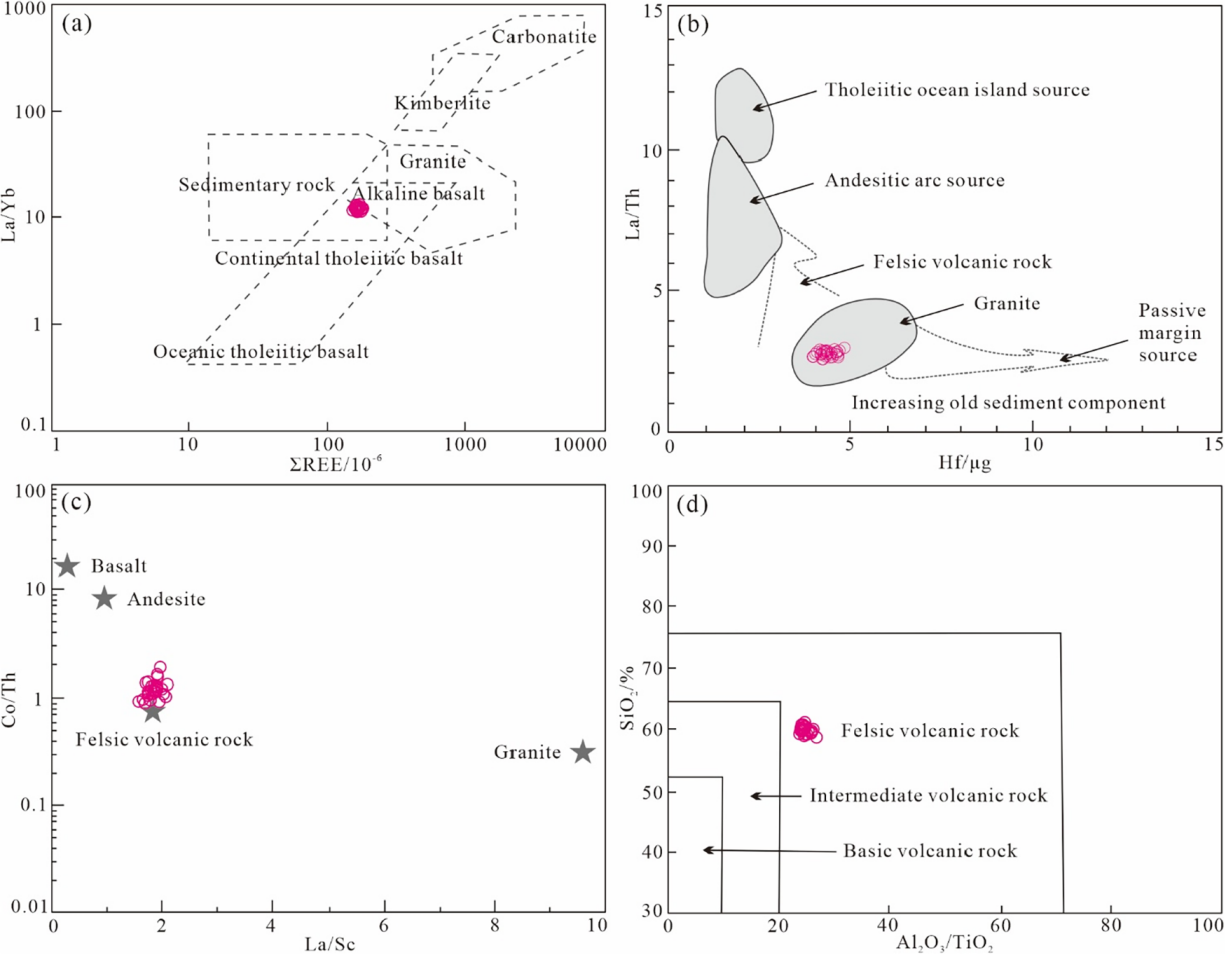
Sediment provenance analysis of mudstones from the Middle Permian Xueyuanhe Formation in Raggyorcaka area. (a) ∑REE–La/Yb diagram; (b) Hf–La/Th diagram; (c) La/Sc–Co/Th diagram; (d) Al_2_O_3_/TiO_2_–SiO_2_ diagram.

During the Late Paleozoic, as the Paleo-Tethys Ocean continued to subduct, a series of intermediate-acidic magmatic rocks developed along the southern margin of the NQD. These magmatic rocks were mainly composed of andesite, rhyolite, dacite and pyroclastic rock, with a small number of granitic plutons. The geochemical characteristics indicated that they formed under a typical island arc tectonic setting.^73,74^ This conclusion is consistent with the tectonic setting discrimination result of the mudstone samples in this paper, suggesting that these Late Paleozoic intermediate-acidic magmatic rocks might be the main source of the Xueyuanhe Formation mudstones.

#### 5.3.2 Tectonic Setting

Sedimentary rocks formed under different tectonic settings exhibit significantly different geochemical characteristics. In this study, a series of diagrams for major, trace, and rare earth elements were used for comprehensive discrimination, and the results were mutually constrained to obtain a more accurate source area tectonic setting. Studies have shown that the enrichment levels of major elements such as SiO_2_, Al_2_O_3_, K_2_O, Na_2_O, and CaO vary significantly under different tectonic settings. The ratio of these element contents can be used to distinguish the tectonic setting of the source area.^75^ In addition, certain stable combinations of rare earth/trace elements (such as La, Sc, Th, Co, Zr, etc.) are not easily affected by later weathering and sedimentation processes and can better reflect the geochemical characteristics of the source area. Consequently, they serve as effective indicators for identifying tectonic setting.^76,77^

In the SiO_2_/Al_2_O_3_–K_2_O/(Na_2_O + CaO) diagram (Figure 10a), all samples fall within the active continental margin area; in the La/Sc–Ti/Zr diagram (Figure 10b), all samples fall near the continental island arc area; in the La–Th–Sc diagram and the Th–Sc–Zr/10 diagram (Figure 10c,d), all samples fall within the continental island arc area and its vicinity, indicating that the source area of the Xueyuanhe Formation mudstones may be the continental island arc tectonic setting.

**Figure 10:**
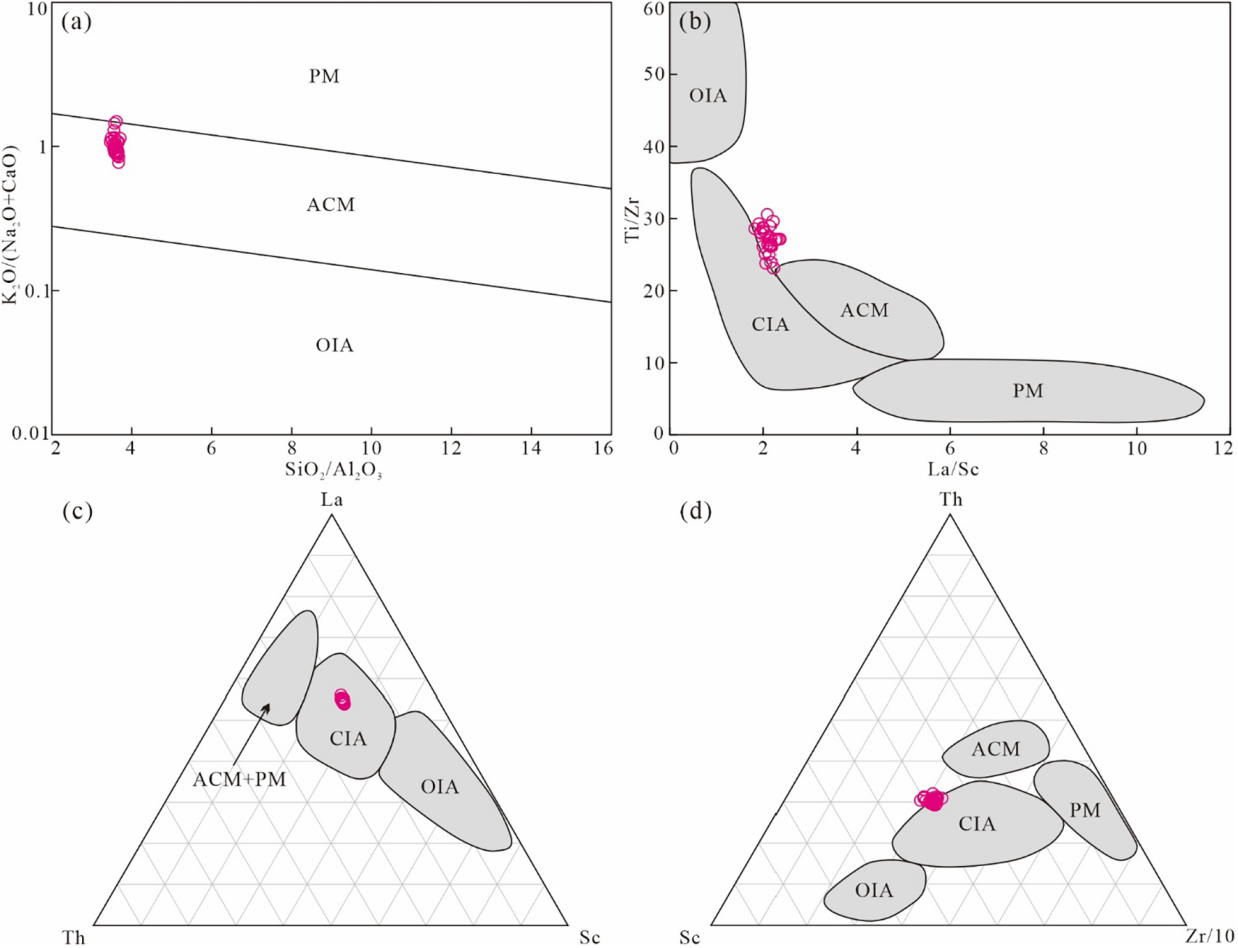
Tectonic setting discrimination diagrams of mudstones from the Xueyuanhe Formation in Raggyorcaka area. (a) SiO_2_/Al_2_O_3_–K_2_O/(Na_2_O + CaO) diagram; (b) La/Sc–Ti/Zr diagram; (c) La–Th–Sc diagram; (d) Th–Sc–Zr/10 diagram. OIA: Oceanic Island Arc; CIA: Continental Island Arc; ACM: Active Continental Margin; PM: Passive Margin.

Based on the geochemical data of the greywackes in eastern Australia, previous researchers systematically summarized the characteristics of rare earth elements in different tectonic settings of sedimentary basins (Table 6), and it has been widely applied.^78–80^ The samples of the Xueyuanhe Formation in the study area are mudstones. Since in the same tectonic setting, the content of rare earth elements in mudstones is about 20% higher than greywackes of the same period, the content of rare earth elements in the mudstone samples is divided by 1.2 to recalculate the relevant parameters.^77,78^ After recalculation and comparison, it was found that the rare earth element characteristics of the mudstone in the study area were almost the same as those greywackes under the background of the continental island arc, indicating that the source area of the mudstone has the tectonic setting characteristics of the continental island arc (Table 6). It should be noted that the tectonic setting discrimination results for sedimentary rocks may be influenced by sediment recycling, grain-size sorting, weathering, and diagenesis. Therefore, interpretation is considered reliable only when combined with regional geological evidence. The analysis of sediment provenance indicates that the sediment source of the Xueyuanhe Formation mudstones may come from the Late Paleozoic intermediate-acidic magmatic rocks along the southern margin of the NQD. These magmatic rocks exhibit a typical continental island arc background, which is consistent with the tectonic discrimination results of this paper. Therefore, it is believed that during the sedimentary period of the Xueyuanhe Formation mudstones, the tectonic activities in the source area were intense, mainly being a continental island arc environment.

**Table 6:** Comparison of Geochemica Parameters between Samples and Different Structural Backgrounds

Tectonic setting	∑REE	LREE/HREE	La/Yb	(La/Yb)_N_	δEu	ω(La)	ω(Ce)
Oceanic island arc	58 ± 10	3.8 ± 0.9	4.2 ± 1.5	2.8 ± 0.9	1.04 ± 0.11	8 ± 1.7	19 ± 3.7
Continental island arc	146 ± 20	7.7 ± 1.7	11 ± 3.6	7.5 ± 2.5	0.79 ± 0.13	27 ± 4.5	59 ± 3.8
Active continental margin	186	9.10	12.50	8.30	0.6	37	78
Passive continental margin	210	8.50	15.90	10.8	0.56	39	85
Mudstone samples	135.20	7.34	12.30	8.82	0.64	30.58	49.58

### 5.4 Mechanism of Organic Matter Enrichment in the Xueyuanhe Formation Mudstones

Organic matter generation, preservation, burial, and transformation all take place within sedimentary basins. Therefore, the sedimentary environment and tectonic setting exert primary control on the development and distribution of source rocks. This paper presents a comprehensive study of the Permian Xueyuanhe Formation mudstones in the NQD, including source rock evaluation, paleoenvironmental analysis, and provenance and tectonic setting discrimination. Furthermore, the organic matter enrichment pattern and its coupling relationship with the sedimentary environment are discussed.

The sedimentary evolution of the Qiangtang Basin was governed by the subduction of the Longmucuo-Shuanghu Paleo-Tethys Ocean (LSPTO). After its opening in the Early Paleozoic, the ocean basin underwent continuous expansion, reaching its maximum size in the Late Paleozoic before initiating northward subduction. This subduction, which began at least in the Late Devonian, persisted until the Middle to Late Triassic, when the ocean basin finally closed and continental collision occurred, marking the transition of the Qiangtang Basin into a unified evolutionary stage.^5,7,81^ During the Middle to Late Permian, the NQD occupied a marine-continental transitional environment. The Raggyorcaka area, situated at a relatively low elevation and experiencing continued marine regression, primarily received coastal sedimentation.^82^ The Xueyuanhe Formation mudstones represent the sedimentary record of this stage (Figure 11a). Other marine-continental transitional facies strata are also present in the Permian successions of the eastern and central-western parts of the basin.^7,8,11,12^

**Figure 11:**
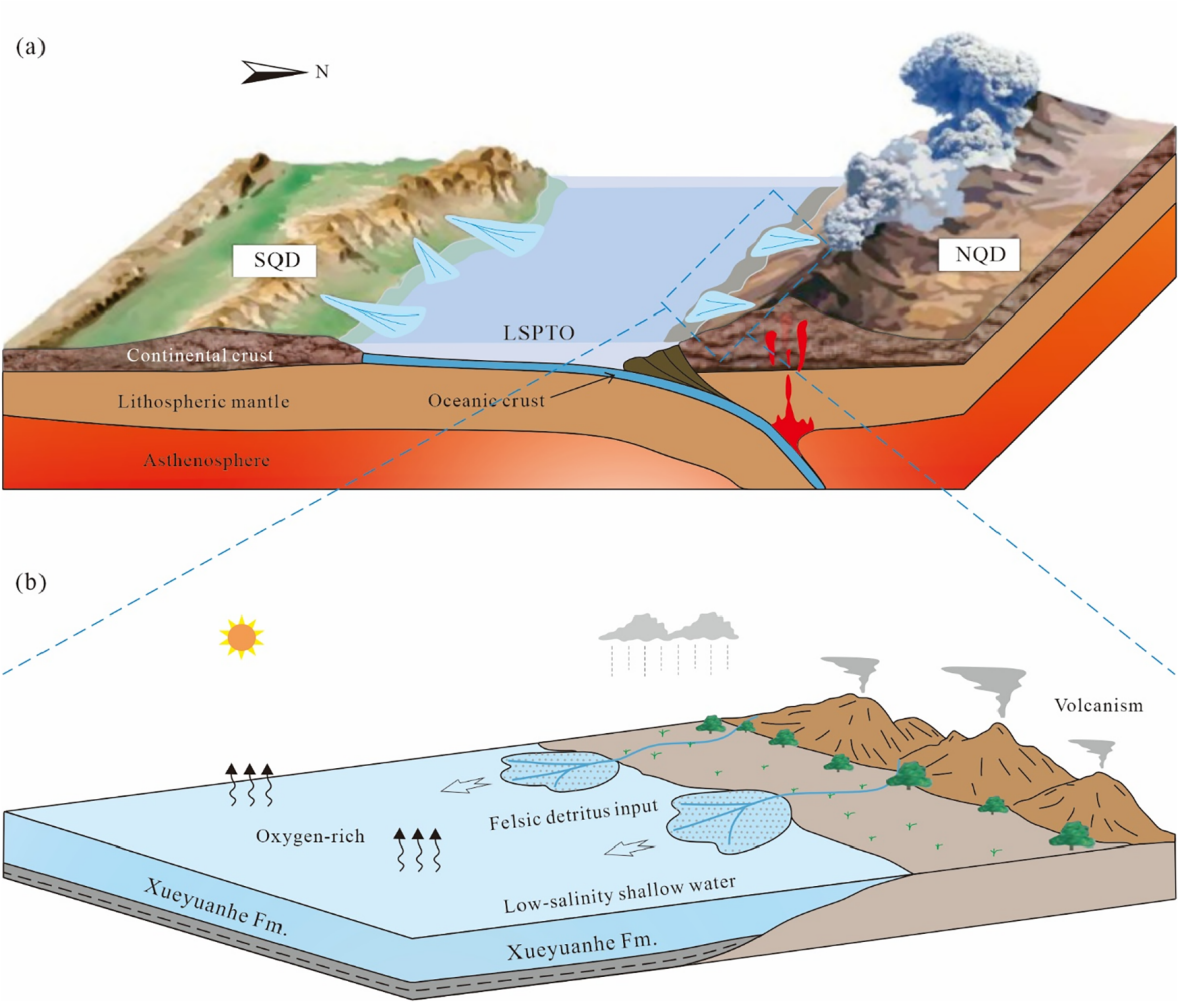
Tectonic setting and sedimentary model of mudstones from the Xueyuanhe Formation in Raggyorcaka area. (a) Tectonic setting of mudstones for the Xueyuanhe Formation; (b) sketch of a sedimentary model for the Xueyuanhe Formation

Paleoenvironmental reconstruction indicates that the Raggyorcaka area experienced warm and humid climatic conditions during deposition of the Xueyuanhe Formation mudstones. Such conditions might have promoted source rock weathering and terrestrial plant growth, thereby enhancing organic matter input during mudstone deposition.^35,36,83,84^ However, continuous marine regression resulted in shallow water and strong hydrodynamic conditions in the Raggyorcaka area. Consequently, frequent oxygen exchange between bottom water and the atmosphere maintained oxic conditions, which facilitated aerobic microbial degradation, the resulting accelerated decomposition of organic matter ultimately limiting its preservation and participation in the sedimentary process. Meanwhile, the high-energy hydrodynamic conditions prevailing during sedimentation caused mechanical disruption of already-deposited organic matter. Suspended organic particles were unable to settle effectively against the turbulent water flow, the rate of organic matter accumulation in the sediment was further diminished. Furthermore, in the context of marine regression, surface runoff became more developed in the Raggyorcaka area. While the overall terrigenous input during deposition was relatively low according to previous studies, the continuous supply of felsic detritus under moderate weather conditions had two important effects: it diluted the organic matter in sediments, while also restricting the supply of essential nutrients to the depositional center. Consequently, primary productivity was suppressed, preventing the development of organic-rich mudstones. Furthermore, the water body maintained consistently low salinity throughout the depositional period. This suppressed the growth of salt-tolerant algae with high productivity potential (such as Cyanobacteria and Dunaliella salina), while the relative contribution of terrigenous higher-plant organic matter increased, resulting in the formation of the organic-lean and humic-type mudstones of the Xueyuanhe Formation (Figure 11b).

In conclusion, preservation conditions were the primary factor controlling organic matter enrichment in the Xueyuanhe Formation mudstones. Against the background of continuous marine regression, the sedimentary water body in the study area was relatively shallow and characterized by low salinity. Despite warm and humid climatic conditions, moderate weathering in the source area led to persistent input of felsic terrigenous detritus. Additionally, the water body remained consistently oxic, accelerating organic matter decomposition. Primary productivity in the water body was also relatively low. This combination of unfavorable factors created a chain effect that inhibited organic matter enrichment and preservation, ultimately resulting in the development of organic-poor mudstones in the Xueyuanhe Formation.

### 5.5 Petroleum Geological Implications

Since the conditions of the source rocks in a single section cannot reflect the oil and gas exploration potential of the entire basin, it is necessary to conduct a comprehensive analysis of the Permian source rocks in the Qiangtang Basin. This study conducts a comparative analysis of typical Permian source rocks based on previous research data.

In the eastern part of the NQD, the upper Permian Naiyixiong Formation mudstones have a relatively high proportion of high-quality source rocks (TOC > 2%), and the organic matter type is mainly type II_2_ and type III kerogen. The maturity of organic matter is in the high maturity to overmaturity stage, and the overall hydrocarbon generation potential is good.^11,12^ In the Jiaomuri area in the NQD, the average TOC content of the Zhanjin Formation mudstones reaches 1.15%. The organic matter type is mainly type III kerogen, and the thermal evolution stage is mainly the mature stage.^5,9^ The TOC content of the Xueyuanhe Formation mudstones varies greatly. A few samples have relatively high TOC content (1.21% and 16.60%), which are classified as medium-to-good source rocks.^85^ The TOC content of the Raggyorcaka Formation mudstones ranges from 0.08% to 19.40%. Among them, two samples have TOC content as high as 15.22% and 19.40%, which are good source rocks. The organic matter type is mainly type II_2_, and the maturity of the organic matter is at the high-maturity stage, reaching the stage of generating gas.^85^ All the above studies have shown that the Permian source rocks in the Qiangtang Basin have good potential for hydrocarbon generation. It is worth noting that due to the limited outcrops of the Permian strata in the Qiangtang Basin and their strong weather, continuous investigation and evaluation work still need to be carried out.

The Permian source rocks, as one of the important high-quality source rocks within the oil and gas enrichment area of the Tethys tectonic domain, are the key exploration layers for the future. They are mainly distributed in the coastal areas of the Caspian Sea, Zayangshan, Central Iran, Tarim and Sichuan basins. Among them, the TOC content of the Lower Permian black mudstone in the Pre-Caspian Basin of Central Asia ranges from 0.90% to 7.91%.^86–88^ The TOC content of the Upper Carboniferous-Permian oil shale and mudstone in the Zaysan Basin is from 0.50% to 21.00%.^89^ The TOC content of the Permian mudstone in the Iran Basin is from 3.50% to 18.60%.^90^ The TOC content of the Permian mudstone and oil shale in the Tarim Basin is from 0.40% to 4.50%.^91,92^ The TOC content of the Permian mud limestone in the Sichuan Basin ranges from 0.20% to 1.50%, while the TOC content of the mudstone and shale is from 0.02% to 18.65%.^93,94^ These source rocks have good comparability with the Permian source rocks in the Qiangtang Basin, and they have significant indicative significance for guiding the exploration of oil and gas in the Late Paleozoic strata of the Qiangtang Basin.

## 6 Conclusions

(1) The mudstone of the Xueyuanhe Formation exhibits low organic matter abundance. The organic matter type is mainly type II_2_ kerogen, with a minor amount of type III kerogen. Their organic matter evolution has reached a high to overmature stage of thermal evolution. Collectively, these characteristics indicate poor hydrocarbon generation potential.
(2) Although the Xueyuanhe Formation mudstones were deposited under warm and humid climatic conditions favorable for organic productivity, several factors combined to inhibit organic matter enrichment. The turbulent, oxic water body during deposition promoted organic matter decomposition rather than preservation. Additionally, moderate weathering, slow sedimentation rates and continuous supply of felsic terrigenous detritus not only diluted organic matter but also restricted nutrient availability, leading to low primary productivity. Consequently, organic matter accumulation was limited, resulting in the low TOC contents observed in Xueyuanhe Formation mudstones.
(3) The tectonic setting of the source area for the Xueyuanhe Formation mudstones is interpreted as a continental island arc. The sedimentary provenance is dominated by felsic volcanic rocks, with possible minor inputs from basaltic and sedimentary sources. These sediments were likely derived primarily from the Late Paleozoic intermediate-acidic magmatic rocks exposed along the southern margin of the North Qiangtang Depression.
