# Correction to “Geochemical Characteristics and Formation Environment of Mudstones in the Xueyuanhe Formation in the Raggyorcaka Area, Qiangtang Basin”

**DOI:** 10.1021/acsomega.6c08009

**Published:** 2026-09-23

**Authors:** Jie Peng, Yuan Xie, Mei Yue, Qinghua Peng, Zhongwei Wang, Rui Wang, Zhonglin Wang, Yonghong Hu

In the original article, several author affiliation assignments were incorrect. The affiliation assignments for Jie Peng, Yuan Xie, Mei Yue, Qinghua Peng, and Yonghong Hu are corrected. The corrections are reflected in the Author Information of this Correction. These corrections do not affect the scientific content, results, or conclusions of the paper.

**Author Information**

*Corresponding Author*

Mei Yue – Research Center of Applied Geology of China Geological Survey, Chengdu, Sichuan, 610036, China; College of Energy, Chengdu University of Technology, Chengdu, Sichuan 610059, China; Email: yuemei6035@163.com

*Authors*

Jie Peng – Research Center of Applied Geology of China Geological Survey, Chengdu, Sichuan, 610036, China; orcid.org/0009-0007-2044-9349

Yuan Xie – Research Center of Applied Geology of China Geological Survey, Chengdu, Sichuan, 610036, China

Qinghua Peng – Research Center of Applied Geology of China Geological Survey, Chengdu, Sichuan, 610036, China; orcid.org/0000-0002-3569-188X

Zhongwei Wang – School of Geoscience and Technology, Southwest Petroleum University, Chengdu, Sichuan 610500, China

Rui Wang – Oil & Gas Survey Center of China Geological Survey, Beijing 100083, China

Zhonglin Wang – College of Energy, Chengdu University of Technology, Chengdu, Sichuan 610059, China

Yonghong Hu – Research Center of Applied Geology of China Geological Survey, Chengdu, Sichuan, 610036, China

